# Still standing: Recent patterns of post-fire conifer refugia in ponderosa pine-dominated forests of the Colorado Front Range

**DOI:** 10.1371/journal.pone.0226926

**Published:** 2020-01-15

**Authors:** Teresa B. Chapman, Tania Schoennagel, Thomas T. Veblen, Kyle C. Rodman

**Affiliations:** 1 The Nature Conservancy, Boulder, Colorado, United States of America; 2 Department of Geography, University of Colorado Boulder, Boulder, Colorado, United States of America; 3 Institute of Arctic and Alpine Research (INSTAAR), University of Colorado Boulder, Boulder, Colorado, United States of America; USDA Forest Service, UNITED STATES

## Abstract

Forested fire refugia (trees that survive fires) are important disturbance legacies that provide seed sources for post-fire regeneration. Conifer regeneration has been limited following some recent western fires, particularly in ponderosa pine (*Pinus ponderosa*) forests. However, the extent, characteristics, and predictability of ponderosa pine fire refugia are largely unknown. Within 23 fires in ponderosa pine-dominated forests of the Colorado Front Range (1996–2013), we evaluated the spatial characteristics and predictability of refugia: first using Monitoring Trends in Burn Severity (MTBS) burn severity metrics, then using landscape variables (topography, weather, anthropogenic factors, and pre-fire forest cover). Using 1-m resolution aerial imagery, we created a binary variable of post-fire conifer presence (‘Conifer Refugia’) and absence (‘Conifer Absence’) within 30-m grid cells. We found that maximum patch size of Conifer Absence was positively correlated with fire size, and 38% of the burned area was ≥ 50m from a conifer seed source, revealing a management challenge as fire sizes increase with warming further limiting conifer recovery. In predicting Conifer Refugia with two MTBS-produced databases, thematic burn severity classes (TBSC) and continuous Relative differenced Normalized Burn Ratio (RdNBR) values, Conifer Absence was high in previously forested areas of Low and Moderate burn severity classes in TBSC. RdNBR more accurately identified post-fire conifer survivorship. In predicting Conifer Refugia with landscape variables, Conifer Refugia were less likely during burn days with high maximum temperatures: while Conifer Refugia were more likely on moister soils and closer to higher order streams, homes, and roads; and on less rugged, valley topography. Importantly, pre-fire forest canopy cover was not strongly associated with Conifer Refugia. This study further informs forest management by mapping post-fire patches lacking conifer seed sources, validating the use of RdNBR for fire refugia, and detecting abiotic and topographic variables that may promote conifer refugia.

## Introduction

The number of large wildfires in the western US has increased in recent decades [1]. Weather conducive to large wildfires in forests has also become more extreme and more common over the past few decades due to anthropogenic climate change [2], and a trend toward larger, more severe fires is expected with further warming [3,4]. Larger fires tend to have larger patches of high-severity fire, leaving contiguous expanses without surviving trees [4,5]. In combination with these trends, many dry coniferous forests of the western US with a historically low-severity, high-frequency fire regime also show a marked increase in tree density over the 20^th^ century, increasing the potential for high-severity fires [6–11]. These spatial and temporal trends in wildfire are particularly alarming when combined with warm and dry climatic conditions unfavorable to tree seedlings, resulting in a marked reduction in post-fire forest regeneration [12–14]. The current and projected increase in large fires and lowered capacity to recover may drive transitions of forests to grasslands or shrublands (alternative states) [15]. Identifying indicators of potential shifts in states is critical to understanding forest resilience, defined as the system’s ability to absorb a disturbance and not fundamentally shift to another state governed by a different set of processes [16]. Disturbance legacies, such as individuals that survive and persist in the landscape following a disturbance, leave valuable material like seeds and microsites for disturbance recovery [17]. A better understanding of conditions that influence disturbance legacies is necessary to evaluate potential forest resilience to wildfire.

In response to growing concerns about potential declines in forest resilience, the study of post-fire recovery and landscape legacies has grown rapidly [12,18,19]. For some forest species, the survival of individuals in a fire (resistance) is a critical component of recovery from the disturbance (resilience) [20]. This can be especially important for obligate-seeding tree species dependent on wind or animal dispersal from live trees, such as ponderosa pine (*Pinus ponderosa*), a widespread fire-adapted species in the western US. Following fire, ponderosa pine seedlings are most abundant at distances less than 50 meters from a seed source, limiting natural post-fire regeneration in large patches without seed sources and/or favorable microsite conditions [21–23,13,24–28]. From the Southwest to the northern Rocky Mountains, trends documenting the recent lack of post-fire regeneration have been particularly notable in ponderosa pine forests, due in part to limited post-fire surviving canopy [21,22,24,26,13,28]. For example, in 15 recent (1988–2010) fires occurring throughout southern Colorado and northern New Mexico, an estimated 42% of the total area burned has post-fire ponderosa pine seedling densities below the lowest historical tree densities reported for these forest types [28]. Similarly, 70% of the large Hayman fire in Colorado was predicted to have limited conifer regeneration [22]. In ponderosa pine forests, forested fire refugia (trees that survive fires) are important disturbance legacies that provide seed sources and modify microsite conditions, thereby playing a crucial role in post-fire regeneration and disturbance recovery [19].

In an early description of forested fire refugia, Camp et al (1997) describe areas that “by virtue of topographic position, soil type, or a combination of environmental conditions and vegetation attributes are less frequently affected by disturbances than the surrounding landscape.” These fire refugia are typically unburned or minimally affected by fire and provide valuable resources for post-fire recovery [19]. Locations of fire refugia are influenced by both predictable and stochastic factors, creating both persistent and ephemeral refugia [19]. Local topography, such as cold air drainages, rugged terrain, and mesic topographies have been shown to increase the likelihood of fire refugia [29–31]. Edaphic factors, such as clay and sand content of soils, are known to support variable historic tree densities in the southwestern ponderosa pine forests and, therefore, we hypothesized may also promote sites of fire refugia [32]. However, landscape factors show less influence in creating fire refugia under more severe burning weather [30,31]. Identifying sites with an increased chance of forested fire refugia may help the design of fuel treatments aimed at increasing forest resilience to wildfire under moderate and severe fire weather [33]. At a broad scale, the proportion of fire refugia may not show a declining trend in the last three decades in response to growing fire size and severity [34]. However, analyses of the spatial characteristics of fire refugia and high-severity fire have generally relied on 30-m resolution data from Monitoring Trends in Burn Severity (MTBS) or other 30-m resolution Landsat satellite-derived burn severity indices [5,30,34,13,4], which may be of only limited use when identifying individual trees. Therefore, due to the limitations of the data resolution it remains unknown if high-severity patches contain individual trees as fire refugia [19].

MTBS datasets, such as thematic burn severity classes (TBSC) and the Relative differenced Normalized Burn Ratio (RdNBR), are commonly used to study the trends and impacts of large (≥404 ha) fires in the United States. TBSC use a combination of the Differenced Normalized Burn Ratio (dNBR) and subsequent manual adjustments to classify burn perimeters into thematic burn severity classes including unburned, low, moderate, high, and increased greenness [35]. RdNBR was developed to account for differences in pre-fire vegetative biomass and fuel heterogeneity, which are not fully accounted for in dNBR calculations and are a potential source of bias in dNBR-based burn severity classes [36]. It is important to evaluate the potential of these commonly used MTBS data to identify forested fire refugia and broad-scale trends in post-fire seeds sources, and to assess their utility as a management tool for predicting post-fire recovery and prioritizing areas for reforestation.

Forested fire refugia may be particularly critical in evaluating the resilience of ponderosa pine-dominated forests in the Southern Rocky Mountains to large wildfires. However, the extent, characteristics, and predictability of ponderosa pine fire refugia are largely unknown and understudied. Using fine-scale 1-m aerial imagery, we identified post-fire Conifer Refugia (areas with conifers that survive fire) in 23 wildfires occurring from 1996 to 2013 in ponderosa pine-dominated forests of the Colorado Front Range (CFR) and contrast it to the opposite condition, Conifer Absence (absence of post-fire conifer survivorship). We evaluate post-fire Conifer Refugia in terms of: 1) their spatial characteristics, 2) how reliably 30-m resolution MTBS databases (TBSC and RdNBR) detect their spatial variation within fire perimeters, 3) and which biotic and abiotic landscape variables influence and best predict their presence. We hypothesized that in addition to pre-burn forest cover, abiotic factors such as soils, moister topographies, and proximity to streams may have an important role in providing post-fire refugia across the CFR. We further hypothesized that these factors would have less importance in predicting post-fire survivorship under extreme fire weather conditions.

## Methods

### Study area

**Fig 1:**
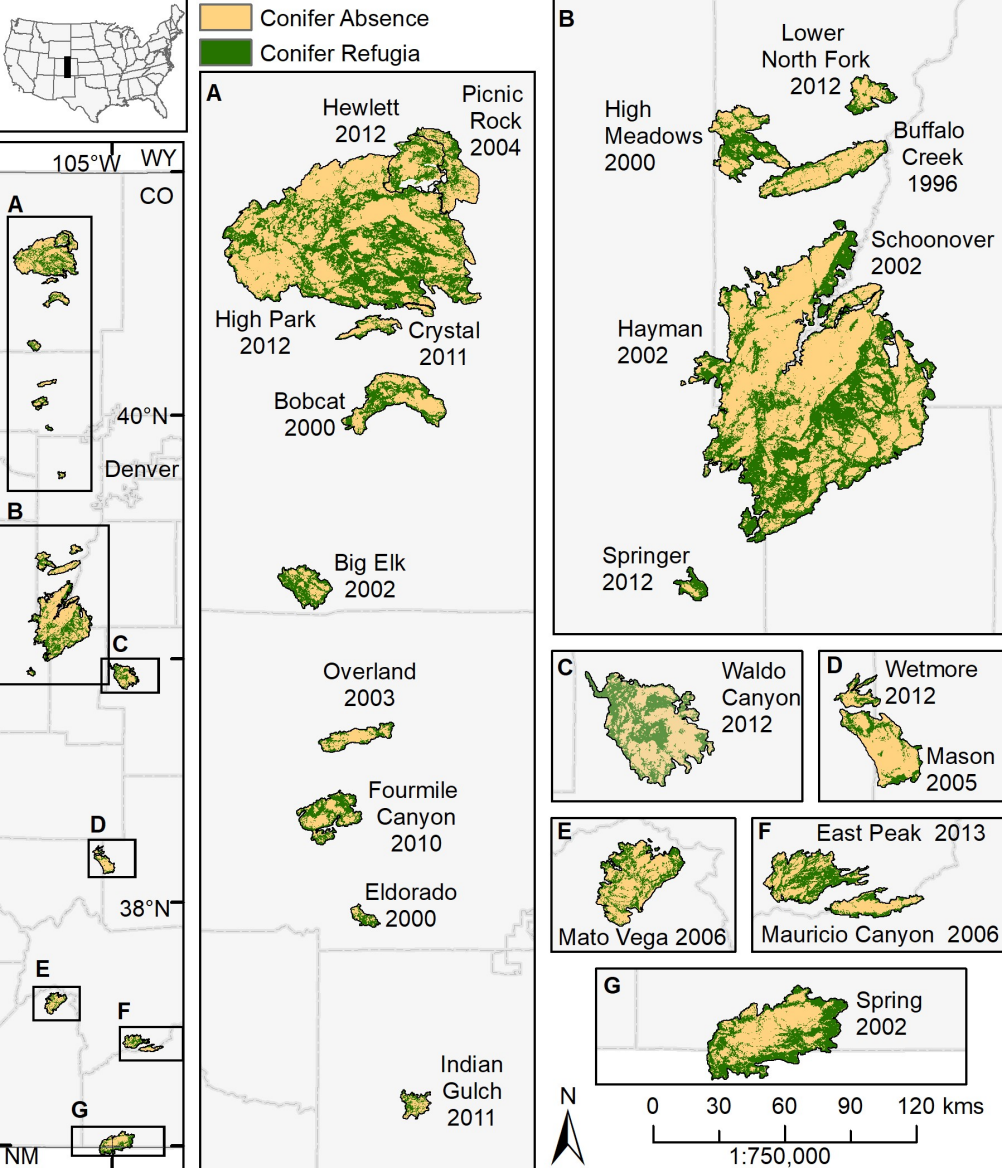
Overview of the study area encompassing 23 wildfires that burned throughout ponderosa pine-dominated forests in the Colorado Front Range from 1996–2013. We used systematic aerial image interpretation of 2015 NAIP imagery to identify post-fire presence (Conifer Refugia) or absence (Conifer Absence) of at least one mature conifer in each 30-m grid cell within each fire perimeter. Each fire is mapped using the binary variable Conifer Refugia or Conifer Absence.

We obtained fire perimeters for 23 wildland fires in Colorado east of the continental divide and within the Southern Rocky Mountains Ecoregion from 1996 to 2013 from MTBS [37] (Fig 1). We selected wildfires that had: 1) greater than 50% of the pre-fire vegetation type classified as forests or woodlands with a prevalence of cover types in ponderosa pine (*Pinus ponderosa* var. *scopulorum*) and montane mixed conifer forests, based on 2001 LANDFIRE existing vegetation types (S1 Table), and 2) less than 50% of the fire area within the Wildland Urban Interface or Intermix [38]. For the 1996 Buffalo Creek fire, we estimated pre-fire vegetation types by characterizing the 2001 LANDFIRE existing vegetation types within an equivalent area outside the fire perimeter (1000-m ring buffer). Of the total area within all fire perimeters, 42.7% was classified as Southern Rocky Mountain Dry-Mesic Montane Mixed Conifer Forest and Woodland and 27.0% was Southern Rocky Mountain Ponderosa Pine Woodland (S2 Table). Forest vegetation types covered 91.2% of the total area within fire perimeters and individual fires averaged (± SD) 88.3 ± 10.9% forested vegetation types. Most fires are suppressed aggressively throughout the CFR, yet under typical warm conditions with dry winds, fires often escape initial containment [39]. All 23 fires were declared FEMA Disasters, ranging from Fire Suppression Authorization to Major Disaster Declaration (FEMA), and one fire was an escaped prescribed fire (Lower North Fork 2012). Peak fire season in the Southern Rockies occurs between June and September [40]. Fires in the analysis occurred between late March through late October, with a median daily burn date occurrence of mid-July (Julian day 194). Fires spanned 1588 m to 3462 m in elevation (mean 2296 ± 238 m), covering the elevation zones of the lower and upper montane in the CFR.

The CFR generally experiences a continental climate, with extreme diurnal and seasonal fluctuations in temperature, and dominant westerly winds [41]. Based on 30-year climate normals from 1981–2010, areas within fire perimeters experienced mean (range) annual precipitation of 51.2 cm (41.6 to 70.0), a minimum annual temperature of -1.04°C (-3.6°C to 1.4°C), and maximum annual temperature is 14.0°C (10.6°C to 17.5°C) [42]. Yearly and monthly maximum temperatures along the CFR have increased from 1953 to 2008. At lower elevations, these increases have been most pronounced from 1989 to 2008 [43]. Based on monthly PDSI (Palmer Drought Severity Index) regional values, the 1996–2013 period of the study included the first (2002), sixth (2012), and 11^th^ (2006) most severe June droughts calculated along the CFR since 1895 [44].

The geographic extent of the CFR is delimited to the east by the grasslands of the Great Plains and expands west to the Continental Divide [41]. In this region, elevation and topographic-moisture gradients drive patterns of forest composition [45]. Along the latitudinal gradient of the study area, elevations for the dominant vegetation zones shift by approximately 150 m [46]. The central lower montane ranges from 1828 m—2438 m, where ponderosa pine is dominant or co-dominant with Douglas-fir (*Pseudotsuga menziesii* var. *glauca*), especially on more mesic sites [46]. On drier sites and at lower elevations, ponderosa pine can form more open woodlands (tree cover >20% and ≤ 40%) and savannas (tree cover ≤ 20%), as well as in the lower ecotone where grasslands transition to forests between 1676 m-1828 m [46]. In the upper montane zone, at higher elevations and with higher moisture availability, ponderosa pine and Douglas-fir mix with subalpine species such as lodgepole pine (*Pinus contorta* var. *latifolia*), subalpine fir (*Abies lasiocarpa*), Engelmann spruce (*Picea engelmannii*), and aspen (*Populus tremuloides*) to form naturally dense stands [46]. In the southern portion of the study area, ponderosa pine mixes with the shrub form of Gambel oak (*Quercus gambelli*) [47]. Soils are typically shallow and coarse with the best developed profiles in larger valleys bottoms, and consist of sandy loams or loamy sands [41].

Mixed stands of coniferous and/or angiosperm trees with ponderosa pine are expected to vary in post-fire regeneration depending on species-specific regeneration processes and gradients of fire severity and climate [28,48]. Sprouting woody shrubs, such as Gambel oak, and suckering aspen stands have shown areas of abundant post-fire regeneration in our study region [28,49–51]. Favorable sites, availability of post-fire seed sources, and climatic conditions have a strong influence on the successful establishment of post-fire obligate-seeding species in the western US and the Southern Rocky Mountains [28,48,51,52]. Lodgepole pine can exhibit abundant post-fire seedlings where serotinous cones (fire-adapted canopy seed banks) are present, and has consequently shown less dependence on distance to seed source than other conifers in dry mixed conifer forests [12,23,51]. Douglas-fir has wind dispersed seeds and depends on proximity to seed sources and shade-providing canopy cover for successful post-fire regeneration [22,28,28,51]. Post-fire regeneration from bird- and animal-dispersed seeds of Rocky Mountain juniper (*Juniperus scopulorum*) and pinyon pine (*Pinus edulis*) tend to be high within post-fire refugia [51]. Although we focus primarily on potential limitations to ponderosa pine regeneration in the montane forests of the CFR in this analysis, similar limitations to post-fire regeneration exist for other obligate-seeding conifer species in the dry montane region [24,28,51].

Fire regimes in ponderosa pine-dominated forests along the CFR also vary by elevation [53,54]. Below 2260m, historically there was a higher proportion of high-frequency, low-severity fire. Above 2260m, mixed- or high-severity fires were more common [6,54]. At stand scales, low-severity fires, as defined by Odion et al. (2014), generally cause mortality to less than 20% of the canopy trees by basal area, primarily burning surface fuels [7], whereas high-severity fires, as defined by Odion et al. (2014), kill greater than 70% of the canopy trees by basal area, burn surface and canopy fuel, and occur under extreme fire weather, such as high winds [7]. At the stand-scale, mixed-severity fires intermix patches of both low- and high-severity fire, and have between 20-70/80% remnant tree canopy [7,55]. MTBS thresholds for Low, Moderate, and High burn severity are subjective, based on analyst interpretation, and do not necessarily reflect these mortality thresholds for burn severity classes. Modern forests of the CFR reflect complex spatio-temporal patterns of human impacts through episodes of intentional burning during severe droughts in the 19^th^ century, 20^th^ century fire exclusion, grazing, and logging [56–58]. In the lower montane zone, the exclusion of frequent low-severity fires has resulted in increased stand densities [6,9–11,59]. In upper montane mixed-conifer forests, synchronous and high density tree establishment following high-severity fires and historical logging make it difficult to isolate the effects of 20^th^ century fire exclusion [6,55,56]. Where Gambel oak and ponderosa pine mix, the fire history is less clear and past land use may have increased Gambel oak densities [47].

### Data processing

**Fig 2:**
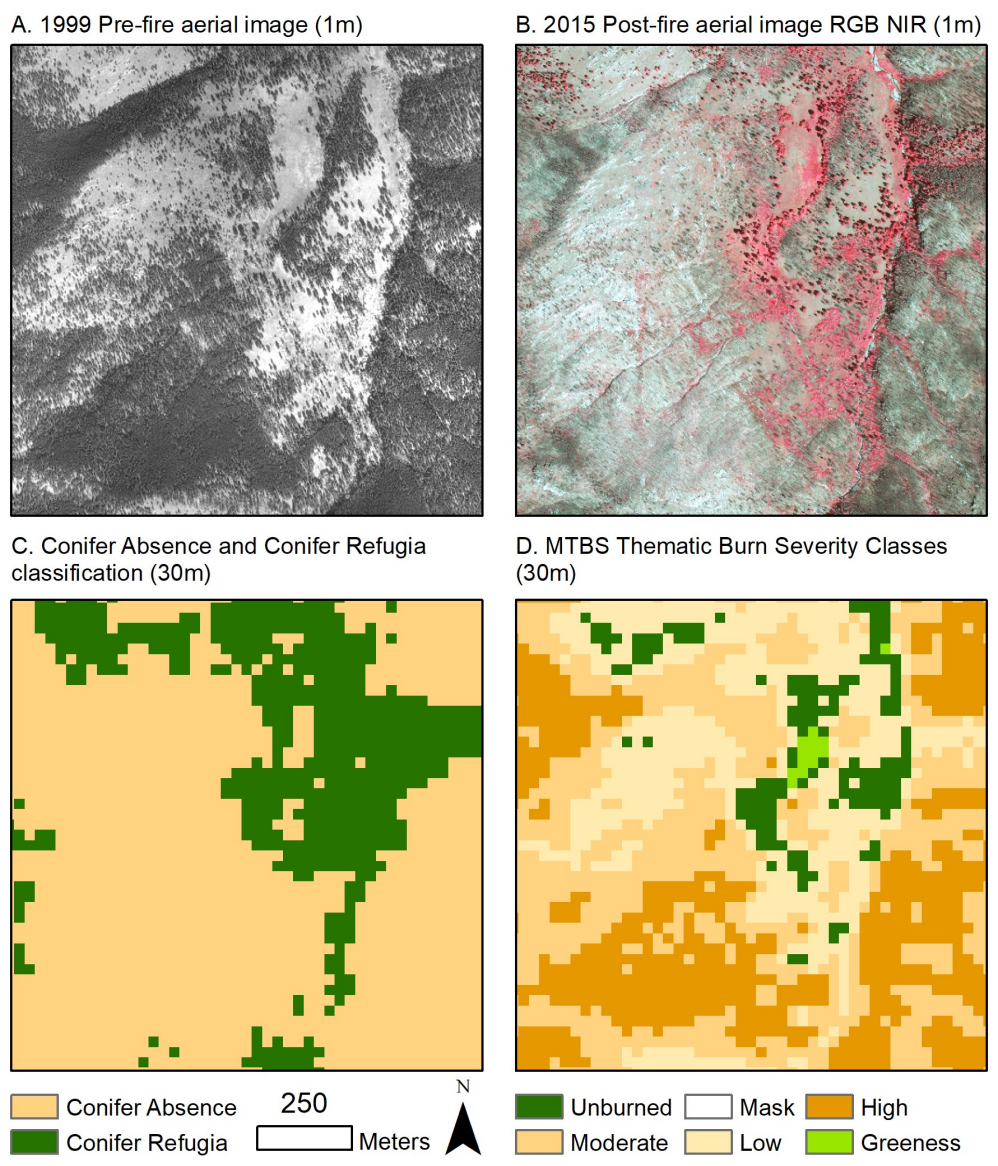
Demonstration of methods used to create binary Conifer Refugia and Conifer Absence variable. Conifer Refugia is defined by the presence of post-fire mature conifers, and Conifer Absence contained no live mature trees following the fire. Panels A-D span the same extent within the 2012 High Park fire. Pre-fire black and white aerial images were used to enhance the classifications of 2001 NLCD Pre-fire Forest Cover (A). Post-fire four band aerial images in 2015 (B) were used to identify presence or absence of a mature post-fire conifer (C) in each 30-m grid cell that was recorded by Monitoring Trends in Burn Severity (MTBS) Thematic Burn Severity Classes (D).

As our dependent variable, we created a binary condition through visual assessment by manually classifying presence or absence of post-fire mature live conifers–Conifer Refugia and Conifer Absence, respectively–within 30-m grid cells using 2015 National Aerial Image Products (NAIP; 1-m resolution, RGB NIR bands) at a scale of 1:4000 (Fig 2). We created grid cells within all fire perimeters by converting the MTBS and GEOMAC raster grids to centerpoints and building a 30-m fishnet surrounding the points. We classified a pixel centerpoint as Conifer Refugia if any visible portion of a live conifer with defined crown morphology with a shadow was present within the 30-m fishnet. We assumed that conifer post-fire regeneration within the fire perimeter would not be detectable at this scale (canopy width narrower than 1m and not casting visible shadows). We visually differentiated between conifers and angiosperm trees and shrubs through the darker canopy, lower near infrared reflectance, and more defined crown morphology of conifers. We acknowledge that tree mortality observable in the 2015 images will be the combined result of death in the fire and any lagged mortality due to other reasons, such as fire damage, post-fire insect attack, or drought stress.

We assessed the accuracy of the Conifer Refugia and Conifer Absence classification with two methods. First, we conducted a visual assessment of 500 randomly generated points for each class using NAIP 2015 imagery. Different analysts conducted the initial classification and the manual accuracy assessment. Conifer Refugia was 98.4% accurate (492/500) and Conifer Absence was 97.8% accurate (489/500) for a total accuracy of 98.1% at the 30-m resolution. We also used field plots from three published studies documenting the presence or absence of mature conifers in recently burned ponderosa pine-dominated forests of the CFR [22,24,28]. We buffered field plot centers by 15 m for Rodman et al. (2019) and Chambers et al. (2016) data and by 25 m for Rother et al (2016) data to account for plot or transect size and to ensure alignment with 30-m pixels. We used field plots uniformly located in Conifer Refugia or Conifer Absence within these buffered distances and excluded field plots that intersected both classes (n = 592 out of 773). Conifer Refugia was 99.5% accurate (203/204) and Conifer Absence was 97.4% accurate (378/388) for a total accuracy of 98.1%. We acknowledge that the field-based accuracy assessment may be biased toward more uniform larger patches.

We determined the closest distance to a potential post-fire seed source by calculating the Euclidean distance from Conifer Absence to surviving trees either: 1) outside the burn perimeter, using the 2014 Landfire Tree Cover (>0%), and 2) within the burn perimeter, defined by Conifer Refugia. We calculated the total area of Conifer Absence within fire perimeters greater than 50m from a potential conifer seed source to estimate the area throughout all fires that is likely to demonstrate low post-fire seedling abundance due to seed source limitations, based on published field observations [22,24]. For a comparison of pre- and post-fire distributions, we also determined the closest distance to a potential pre-fire seed source by calculating the distance to Pre-fire Forest Cover >0% within each fire.

To assess which factors best predicted the presence of post-fire Conifer Refugia, we generated 25 landscape predictor variables at a 30-m resolution based on the following broad categories: 1) abiotic environment, 2) biotic environment, 3) daily fire weather, 4) anthropogenic influence, and 5) fire event influence (Table 1). Data sources of predictor variables and rationale for expected relationships are provided in a supplemental table (S3 Table). To generate daily fire weather variables, we determined burn dates for each pixel using three sources: 1) Geospatial Multi-Agency Coordination Wildland Fire Support (GeoMAC) Fire perimeters [60], 2) MODIS Burned Area Products [61], and 3) FEMA incident reports [62]. Pre-Fire Forest Cover is based on a modified 2001 National Land Cover Database (NLCD) tree canopy cover layer and is the percent of tree cover in a 30-m grid. Detailed methods used in creating daily fire weather and Pre-Fire Forest Cover variables are described in supplemental text (S1 Text). We did not remove non-forested pixels from our analysis for the following reasons: 1) 91.5% of the area within fire perimeters was classified as a forest vegetation type at a 30-m resolution, 2) 95% of the area within fire perimeters had the presence of tree cover according to our Pre-Fire Forest Cover, and 3) two percent of the Conifer Refugia was mapped in areas classified as 0% Pre-fire Forest Cover. Layers were aligned and projected to NAD 1983 Albers Equal Area prior to analysis. To further refine Pre-fire Forest Cover, we removed regions classified as developed, open water, or agriculture from the 2011 NLCD and home locations from a regional WUI layer [63]. Since we used a single layer for Pre-fire Forest Cover for all fires, we removed areas where this variable would have been modified by previous fires or fuel mitigation treatments (thinning). We removed areas within 200 m of overlapping burn perimeters (twice burned areas) and all grid cells 30 m from the fire perimeter edge. We also removed areas within fuel mitigation projects (i.e. any management activity that potentially altered either pre- or post-fire tree cover canopy) as delineated by the LANDFIRE public events 2014 layer, the US Forest Service (USFS) Forest Service Activity Tracking System (FACTS) database, and a recent compilation of Front Range Fuel Treatments [64]. In an overlay of these fuel treatment databases and the 23 fire perimeters, three percent (4511 ha) of the total burned area had been treated with mechanical thinning and/or prescribed fire fuel treatments prior to burning and was removed from the analysis. We included anthropogenic variables (Distance to Roads and Distance to Homes) to account for some of the influence of fire suppression activities in these fire perimeters. We acknowledge that we were not able to account for all fire suppression activities, which may also occur in more remote areas and can influence tree survivorship. We also included the categorical variable Fire, as many variables (i.e., soil type, fire weather) are likely to covary with other fire-level effects that were not included in our analyses.

**Table 1:** List of 25 landscape predictor variables generated for the random forest model meant to classify Conifer Refugia and Conifer Absence. An expected positive relationship between the variable and Conifer Refugia is denoted with (+) and an expected negative relationship with (-).

Variable name, inclusion in model (*), and expected relationship with Conifer Refugia (- or +)	Variable Definition
Fire weather variables	
1. Maximum Temperature* (-)	Daily maximum temperature on burn date
2. Maximum Wind Speed (-)	Daily maximum wind speed on burn date
3. Minimum Relative Humidity (-)	Daily minimum relative humidity on burn date
4. Fire Danger Rating (-)	Class rating based on Burning Index and Energy Release Component and local station manager input, interpolated at 10km grid between stations.
Anthropogenic influence variables	
5. Distance to Homes* (+ or -)	Euclidian distance (m) to identified home location (point).
6. Distance to Roads* (+)	Euclidian distance (m) to identified home location (point).
Biotic variables	
7. Pre-Fire Forest Cover* (+ or -)	Pre-fire percent canopy tree cover, as determined by a modified 2001 NLCD tree cover layer in 30-m grid cell.
8. Pre-Fire Distance to Savanna* (-)	Euclidian distance (m) to Pre-Fire Forest Cover 20% or less.
Abiotic variables	
9. Cost to Streams Order > = 4* (-)	Cost of travelling across terrain slope from a stream centerline greater than or equal to Strahler stream orders 2–4, and with all streams.
10. Cost to Streams Order > = 3* (-)	
11. Cost to Streams Order > = 2* (-)	
12. Cost to Streams (-)	
13. Height Above the Nearest Drainage* (HAND) (-)	DEM normalized using the nearest drainage classification, as created by Donchyts et al. (2016) [65].
14. Compound Topographic Index (CTI) (+)	Wetness index and function of slope and the upstream contributing area.
15. Terrain Roughness* (+ or -)	Standard deviation of elevation in a 7.3 ha rectangular neighborhood (9x9 grid cells).
16. Heat Load Index* (HLI) (+)	A combination of latitude, slope, and aspect that estimates solar radiation on terrain, equation in McCune And Keon (2002) [66]
17. Aspect* (+)	Ranges from 0 (northeast)-2(southwest) (-1 Flat), equation in McCune and Keon (2002) [66].
18. Slope* (+ or -)	Slope in degrees
19. Landforms* (+ or -)	15 unique classifications of landforms based on topographic position, moisture accumulation, and solar radiation, as described by Theobald et al (2015) [67].
20. Soil Max. Percent Clay Content 0-5cm* (-)	Predicted clay content in top 0–5 cm of soil based on USDA SSURGO, as described by Chaney et al (2016) [68].
21. Soil Max. Percent Silt Content 0-5cm* (+)	Predicted silt content in top 0–5 cm of soil based on USDA SSURGO, as described by Chaney et al (2016) [68].
22. Soil Available Water Capacity 0-5cm* (+)	Predicted available water for plants between field capacity and the wilting point based on the USDA SSURGO, as described by Chaney et al (2016) [68].
23. Soil Max. Percent Sand Content 0-5cm* (-)	Predicted sand content in top 0–5 cm of soil based on USDA SSURGO, as described by Chaney et al (2016) [68].
Fire variables	
24. Fire*	Unique Fire Name
25. Daily Area Burned	Patch size of daily area burned within each fire

### Data analysis

#### Spatial characteristics of Conifer Refugia and Conifer Absence

We quantified the spatial patterns for Conifer Refugia and Conifer Absence using several simple metrics including: percent of total fire, maximum patch size, total number of patches, number of small patches (≤0.36 ha [four 30-m pixels]), and percent area in small patches (≤0.36 ha). We determined connectivity of Conifer Refugia and Conifer Absence patches by grouping contiguous pixels of the same classification using an eight-cell neighborhood (pixels that are directly adjacent and diagonal to each other). To determine whether the proportion of Conifer Refugia within fires changed over the study period, we analyzed the percent area of Conifer Refugia in each fire and used ordinary least squares regression to test the significance of a temporal trend 1996–2014 (α = 0.05) [69]. To determine if larger fires have larger patches of Conifer Absence, we tested the presence of a significant relationship (α = 0.05) between the log of fire size and the log of maximum patch size of Conifer Absence, also using a linear least-squares model in R [69]. Values were log-transformed to account for the skewed distributions of fire and patch size. For ease of interpretation, we also present predictions from an untransformed model. We calculated the distribution of distances from Conifer Absence to the closest Conifer Refugia, where longer distances indicate potential areas of post-fire conifer regeneration failure.

#### Predictability of Conifer Refugia using MTBS burn severity metrics

To inform local management plans for potential post-fire recovery programs, we evaluated the reliability of two readily available MTBS databases to represent the locations of post-fire surviving conifers. We calculated the TBSC (unburned, low, moderate, and high) and RdNBR values for pixels of Conifer Refugia and Conifer Absence across all fires. We graphed the percent of Conifer Refugia and Conifer Absence in different thematic burn severity classes. We assessed the power of TBSC and RdNBR to predict Conifer Refugia and Conifer Absence in a Classification and Regression Tree model (CART) in R [69,70]. CART is commonly used in predictive modelling with ecological data because it can handle non-linear relationships, continuous and categorical data, and is easily interpretable [71]. We used 66% of the data as a training set and the remainder was used to test prediction accuracy.

#### Predictability of Conifer Refugia using landscape variables: weather, anthropogenic, biotic, abiotic, and fire factors

To examine factors influencing patterns of Conifer Refugia and Conifer Absence, we first constructed spatial overlays with Pre-fire Forest Cover and daily fire weather variables and compared the distributions in the two classes across equal interval bins of each predictor variable. These descriptive statistics aided in interpretation of subsequent predictive models and associated predictor importance.

To assess how well weather, anthropogenic, abiotic, and biotic factors predict the locations of Conifer Refugia and Conifer Absence, we used Random Forests [72] in R [R Development Team 2018, package ‘randomForest’ [69,73]. We tested multicollinearity among the 25 predictor variables using the “multi.collinear” function in the R package “rfUtilities” and a multi-collinearity threshold of 0.05 [74]. We found no evidence of multicollinearity. To summarize model accuracy, we used the Out-of-Bag (OOB) error estimate, where a lower value indicative of higher predictive accuracy. To assess the stability of Random Forest model predictions, we developed 11 separate models using subsets (20,000 samples in each subset, balanced by class and stratified by fire) of all grid cells (total n ≈1,560,000). We used 500 trees in each model. For each of the 11 distinct model runs, we performed model selection using a comparison of competing models and selected the model that exhibited the lowest OOB error, smallest maximum within class error, and fewest parameters using function “rf.modelSel” in R package “rfUtilities” [74]. To summarize the importance of each predictor across model runs, we present the median and range of variable importance values calculated using the mean decrease in accuracy statistic. Importance values were relativized to sum to one within each model iteration. The 11 models were developed using different data subsets, and thus OOB error is not directly comparable among models. However, for ease of interpretation, we plotted and summarized partial dependence plots of selected variables based on predictions from a primary model (of the original 11), which we selected as the Random Forest model with the lowest OOB error.

## Results

### Spatial characteristics of Conifer Refugia and Conifer Absence

Over 147,000 ha were mapped within the 23 fire perimeters in ponderosa pine-dominated forests of the CFR from 1996–2013 (Table 2). For all fires combined, Conifer Refugia covered 42% and Conifer Absence covered 58% of the total area. Thirty-eight percent of total fire area (> 60,000 ha) was greater than 50m from a potential conifer seed source. Maximum patch size of Conifer Absence was larger (19,640 ha) than that for Conifer Refugia (13,089 ha), although the total number of patches for both was similar (~ 6800). Sixty-nine percent of the Conifer Refugia and Conifer Absence patches were small patches (≤ 0.36ha), covering an extremely small percentage (0.01%) of the total area in each category.

**Table 2:** Spatial metrics of post-fire patches of Conifer Absence (A) and Conifer Refugia (R) within 23 fires that burned ponderosa pine-dominated forests along the Colorado Front Range from 1996–2013. Using image interpretation of 1 m aerial imagery to identify presence or absence of a mature conifer in each 30-m grid cell, Conifer Refugia patches are defined by the contiguous cells with the presence of post-fire mature conifers, and Conifer Absence patches are defined by contiguous cells with the absence of post-fire mature conifers. Median gives the median attribute value across all individual fires and Total gives the attribute value for all fires combined.

	Fire Size			Conifer Absence > 50 m from Conifer Refugia		Maximum Patch Size		Total Number of Patches		Percent of A or R Patches that are small (≤ 0.36 ha)		Percent of A or R Area in Small Patches (≤ 0.36 ha)	
Year Fire	Total (ha)	A (%)	R (%)	A (ha)	A (%)	A (ha)	R (ha)	A	R	A (%)	R (%)	A (%)	R (%)
1996 Buffalo Creek	3911	71	29	2032	52	2695	356	76	298	74	69	0.3	2.9
2000 Bobcat	3645	53	47	1242	34	818	1618	204	175	68	74	1	1.1
2000 Eldorado	392	36	64	48	12	79	233	52	18	62	61	3.1	0.7
2000 High Meadow	3828	47	53	1137	29	808	1724	197	142	63	69	1	0.7
2002 Big Elk	1729	38	62	311	18	405	1044	139	78	65	79	2.3	1.0
2002 Hayman	51977	60	40	23832	46	19631	13050	1990	1999	70	68	0.7	1.1
2002 Schoonover	1103	67	33	517	46	401	122	36	91	75	56	0.5	2.5
2002 Spring	9654	50	50	3380	35	4028	4378	498	335	69	59	1.1	0.7
2003 Overland	1278	62	38	527	41	617	164	64	88	80	67	0.9	2.1
2004 Picnic Rock	3105	61	39	865	24	1223	458	184	471	73	77	1.2	4.3
2005 Mason	4169	73	27	2500	60	2624	550	98	161	68	60	0.3	1.5
2006 Mato Vega	5249	61	39	1896	36	2849	576	334	378	75	71	1.1	2.0
2006 Mauricio Canyon	1730	65	35	868	49	1093	177	78	88	69	53	0.8	1.2
2010 Four Mile Canyon	2321	45	55	562	24	360	1072	182	108	74	79	1.8	1.1
2011 Crystal	1096	61	39	396	4	480	196	56	98	77	67	1.1	2.6
2011 Indian Gulch	641	51	49	95	15	242	272	58	108	66	77	2	3.9
2012 Hewlett	2230	54	46	920	34	875	682	201	150	69	77	1.7	1.7
2012 High Park	34397	59	41	14627	41	10032	9918	1448	1424	68	71	0.7	1.1
2012 Lower North Fork	1361	55	45	495	36	705	225	71	89	73	75	1	1.7
2012 Springer	654	25	75	91	14	141	486	46	8	72	50	3	0.1
2012 Waldo Canyon	8040	52	48	2854	35	2709	2823	376	295	65	64	0.9	0.8
2012 Wetmore	828	57	43	285	34	414	148	59	65	78	55	1.5	1.6
2013 East Peak	4020	44	56	926	23	379	1998	288	145	57	66	1.4	0.7
**Median Fires**	3645	54	46	868	34	875	682	201	150	69	66	1	1.2
**Total Fires**	147423	58	42	60405	38	19640	13089	6739	6823	69	69	0.01	0.01

**Fig 3:**
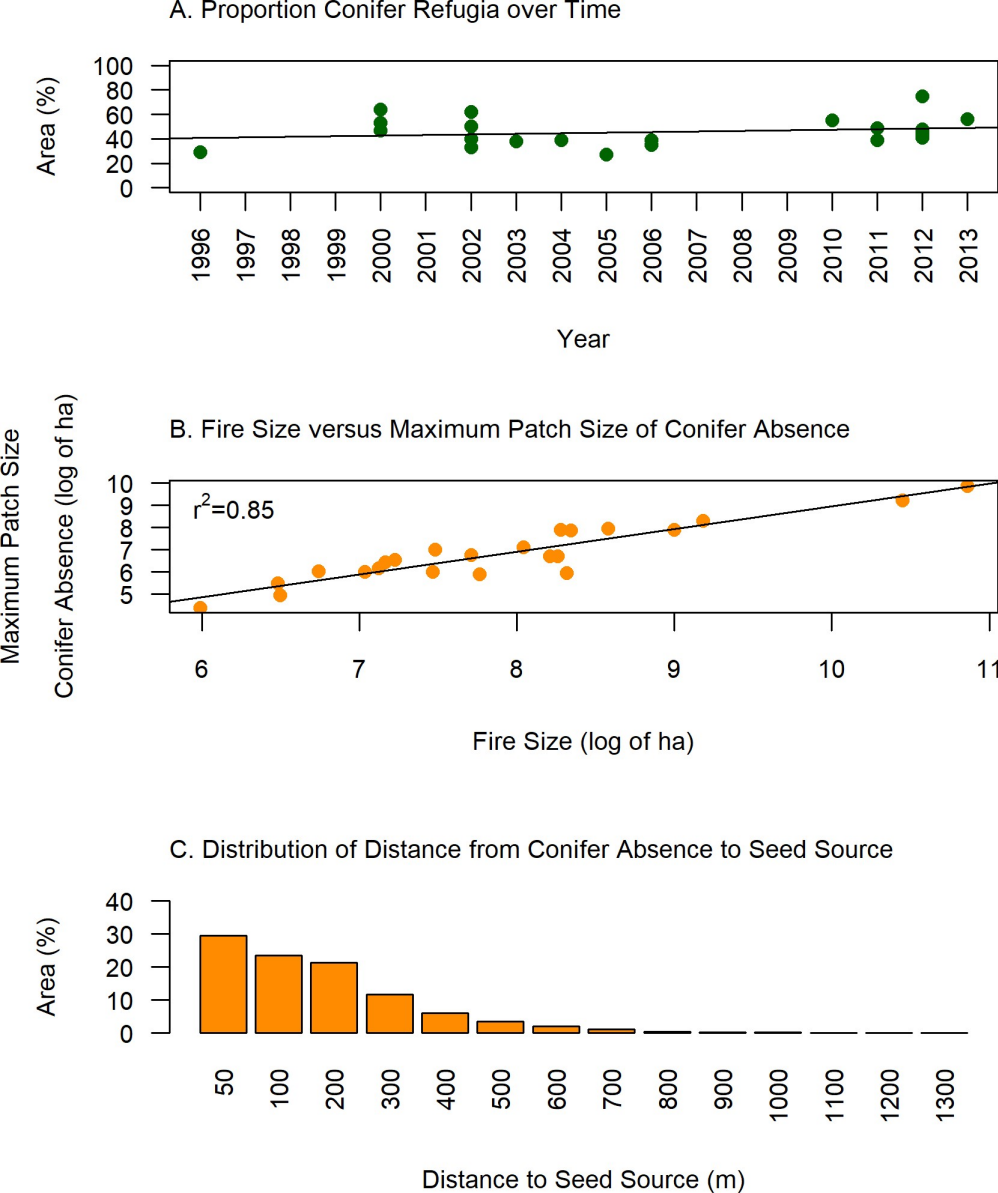
Spatial characteristics of Conifer Refugia and Conifer Absence within 23 fires that burned ponderosa pine-dominated forests along Colorado’s Front Range 1996–2013. A) Percent Conifer Refugia for each fire plotted across time (no significant trend, p≥0.05), B) significant linear relationship between the log of Maximum Patch Size of Conifer Absence and the log of Fire Size (R-squared = 0.85, p≤0.0001), and C) Distribution of Distance from Conifer Absence to Conifer Refugia seed source. Thirty-eight percent of the area within the 23 fire perimeters is greater than 50m from a seed source. Using 1-m aerial image interpretation to identify presence or absence of a mature conifer in a 30-m grid cell, Conifer Refugia is defined by the post-fire presence of at least one mature conifer, and Conifer Absence as the opposite.

The proportion of Conifer Refugia within fire perimeters did not significantly decrease or increase over time between 1996 and 2013 (p ≥0.05, Fig 3A). We found a strong relationship between the log of Maximum Patch Size of Conifer Absence and the log of Fire Size (r^2^ = 0.85, p ≤0.0001, Fig 3B). The slope of the linear model of Maximum Patch Size of Conifer Absence and Fire Size (using untransformed values) was 0.35, indicating that as fire size increased, the largest patch of Conifer Absence in each fire increased in size by roughly a third of the fire size. For example, the predicted Maximum Patch Size of Conifer Absence for a 50,000-ha fire is approximately 13,600 ha. The maximum distance to seed source within Conifer Absence patches extended to 1300 m, but over half was within 200 m (Fig 3C). The average distance to a potential seed source within fires increased from 3.4 (±4.6) m before fires to 60.4 (±32.7) m following fires (S4 Table). The difference in maximum distance to a potential seed source within each fire on average (±SD) increased 434 (±299.5) m between pre- and post-fire measurements. The 2002 Hayman, the 2005 Mason, and the 2012 High Park fires have maximum post-fire distances to potential seed sources greater than 1000 m.

### Predictability of Conifer Refugia using MTBS burn severity metrics

**Fig 4:**
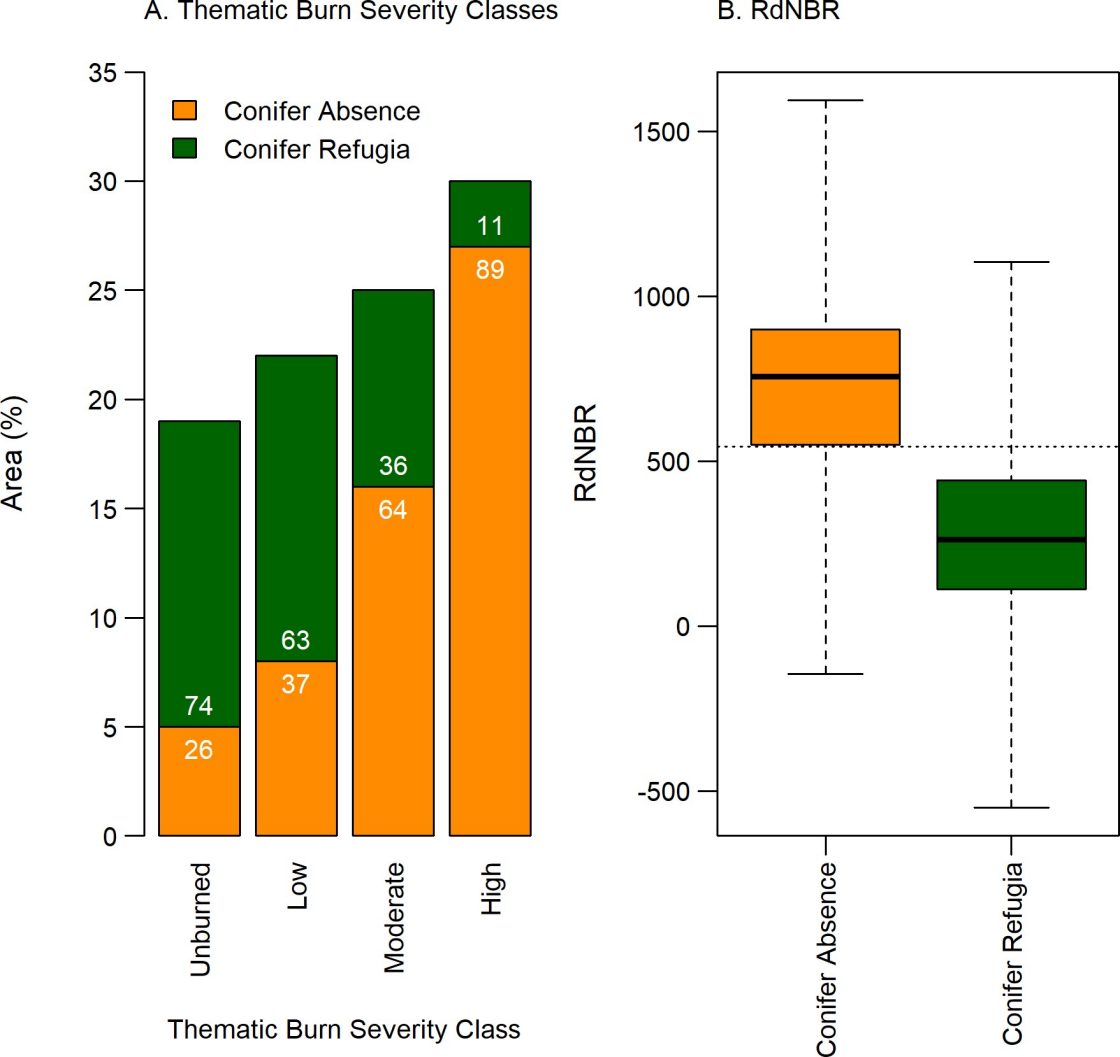
Relationship between Conifer Absence and Conifer Refugia and MTBS burn severity metrics within 21 fires which burned ponderosa pine-dominated forests along Colorado’s Front Range 1996–2013. (A) Thematic Burn Severity Classes (TBSC) as classified by Monitoring Trends in Burn Severity (MTBS) and (B) Relative differenced Normalized Burn Ratio (RdNBR). Using 2015 1m aerial images, we classified Conifer Refugia as the presence of a mature post-fire conifer, and Conifer Absence as the absence of a post-fire mature conifer in 30m grid cells. In (A), Percentage of Conifer Refugia (green) and Conifer Absence (orange) in TBSC is shown within each bar. Horizontal dashed line in (B) denotes the results of a classification and regression tree predicting Conifer Refugia and Conifer Absence using MTBS TBSC and RdNBR, in which a single split at RdNBR <544 best predicted Conifer Refugia and had an overall accuracy of 78%.

**Fig 5:**
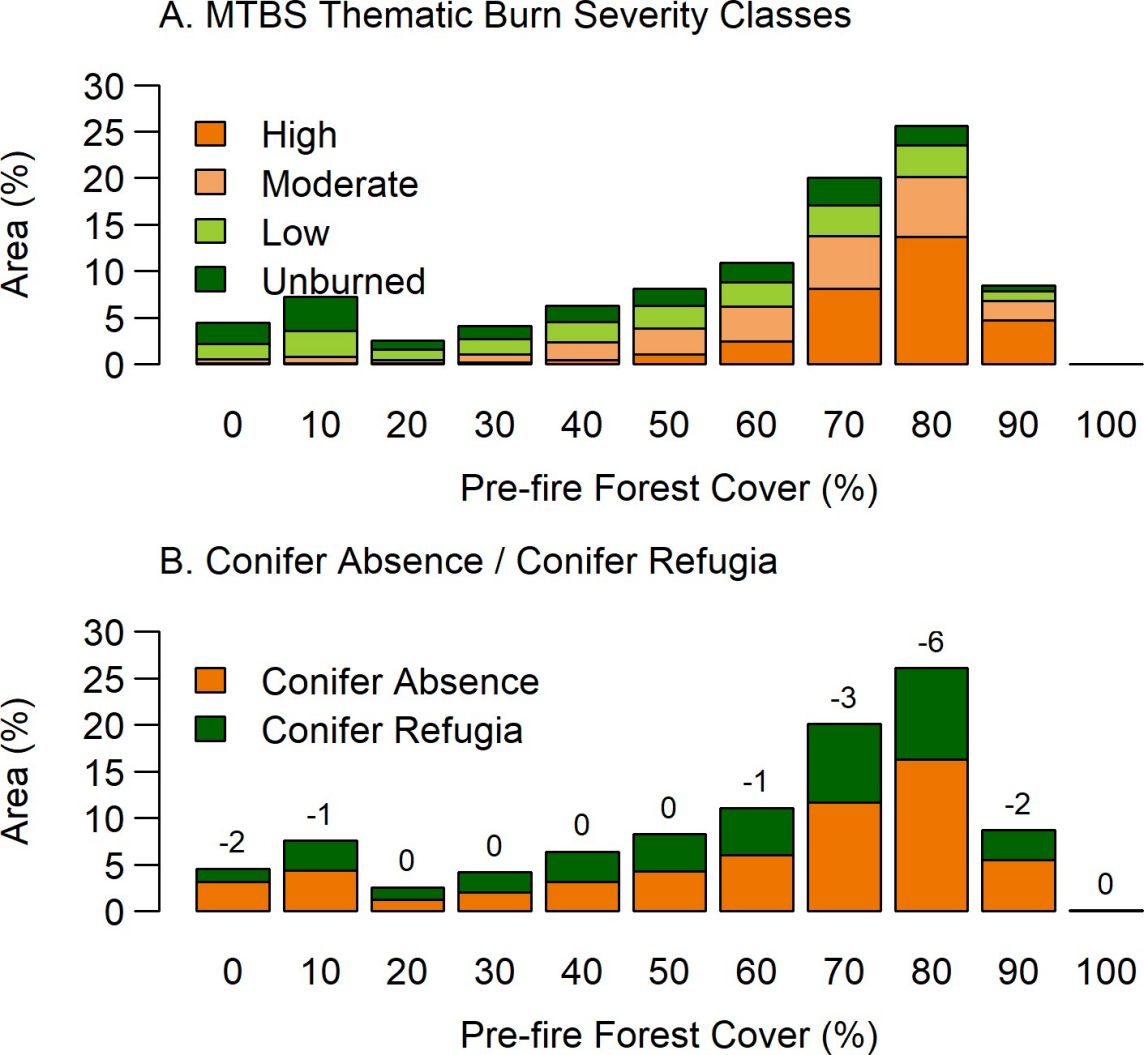
Relationships between forest cover and MTBS thematic burn severity and Conifer Refugia and Conifer Absence. Percent area of (A) Monitoring Trends in Burn Severity (MTBS) thematic burn severity classes (TBSC) and (B) post-fire Conifer Refugia and Conifer Absence with Pre-Fire Forest Cover classes burned by 23 fires in ponderosa pine-dominated forests along Colorado’s Front Range 1996–2013. Pre-Fire Forest Cover was classified by the 2001 NLCD Tree Cover and augmented with 1994 and 1999 aerial imagery. Values above bars in (B) show the percentage point between Conifer Absence and Conifer Refugia (negative numbers denote Conifer Absence is greater than Conifer Refugia).

Both TBSC and RdNBR maps showed relationships with the locations of Conifer Refugia and Conifer Absence across the study area (Fig 4). Within the thematic burn severity classes, High burn severity, which aims to capture areas with substantial canopy mortality had the highest percentage of Conifer Absence (89%), with only 11% of the grid cells classified as Conifer Refugia (Fig 4A). The Moderate burn severity class was comprised of 64% Conifer Absence and 36% Conifer Refugia, and the Low burn severity class was comprised of 37% Conifer Absence and 63% Conifer Refugia. Low and Moderate burn severity classes have a relatively high proportion of Conifer Absence, indicating that these classifications do not always reflect high post-fire conifer survivorship, as is often assumed in less-severe burn classes. Overall, thematic burn severity classes appear to be strongly related to pre-fire forest cover but do not always accurately predict Conifer Refugia, especially in lower forest covers (Fig 5A and 5B). For example, grid cells classified as TBSC High burn severity were less common in areas of lower Pre-fire Forest Cover, yet Conifer Absence comprised approximately half of all areas of Pre-fire Forest Cover <70% (Fig 5B). Specifically, in the 40% Pre-fire Forest Cover bin, half of the area in this class did not have surviving trees, yet TBSC only recorded 7% of the area as High burn severity. A visual example of this incongruence is evident in Fig 2, as MTBS TBSC classify a large open savanna as Low and Moderate burn severity, although it experienced complete canopy mortality. The results of the CART model, using both TBSC and RdNBR as predictors, showed that a single split at RdNBR ≤ 544 best predicted Conifer Refugia and had an overall accuracy of 78% (Fig 4B). RdNBR more reliably predicted Conifer Refugia and Conifer Absence than did MTBS thematic burn severity maps.

### Predictability of Conifer Refugia using landscape variables: weather, anthropogenic, biotic, abiotic, and fire factors

Overlays of fires with Pre-fire Forest Cover reveal that 95% of the total burned area had pre-fire forest cover (>1%), with over half (55%) of the total area having dense pre-fire forests (Pre-fire Forest Cover >70%; Fig 5B). The highest proportion (57%) of post-fire Conifer Absence occurred in areas of dense Pre-fire Forest Cover. However, notably almost 40% of the dense Pre-fire Forest Cover within fire perimeters contained surviving tree cover post-fire. A quarter of the total fire area burned in woodlands and savannas (<40% Pre-Fire Forest Cover) and over half of these more open areas did not have surviving conifers post-fire.

**Fig 6:**
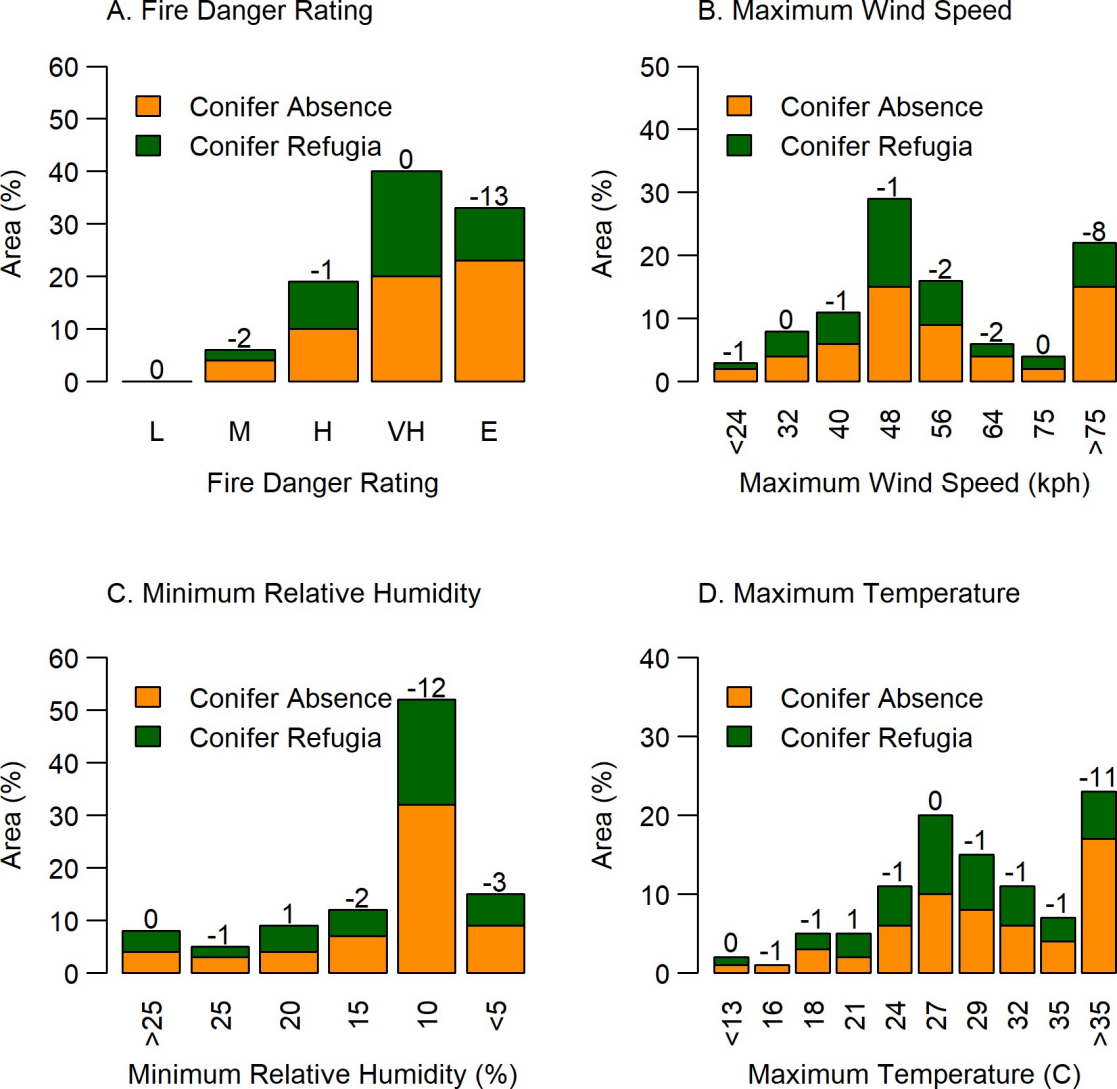
Relationships between weather variables and Conifer Refugia and Conifer Absence. Percent area of Conifer Absence and Conifer Refugia within 23 fires that burned ponderosa pine-dominated forests along Colorado’s Front Range 1996–2013 within climate variable classes for A) Fire Danger Rating (Low, Moderate, High, Very High, Extreme), B) Maximum Wind Speed, C) Minimum Relative Humidity, and D) Maximum Temperature. Values above bars show the percentage point between Conifer Absence and Conifer Refugia (negative numbers denote Conifer Absence is greater than Conifer Refugia).

In contrast to pre-fire forest cover, fire weather strongly influenced the post-fire proportion of Conifer Refugia. Overlays with fire weather variables revealed that burning during moderate fire weather conditions resulted in generally equal proportions of post-fire Conifer Refugia and Conifer Absence (Fig 6A–6D). In contrast, burning during the highest wind speeds, highest temperatures, lowest relative humidity, and most extreme fire danger ratings, coincided with the lowest proportion of Conifer Refugia and highest proportion of Conifer Absence, creating more than a 10% difference in the proportions of the two classes.

**Fig 7:**
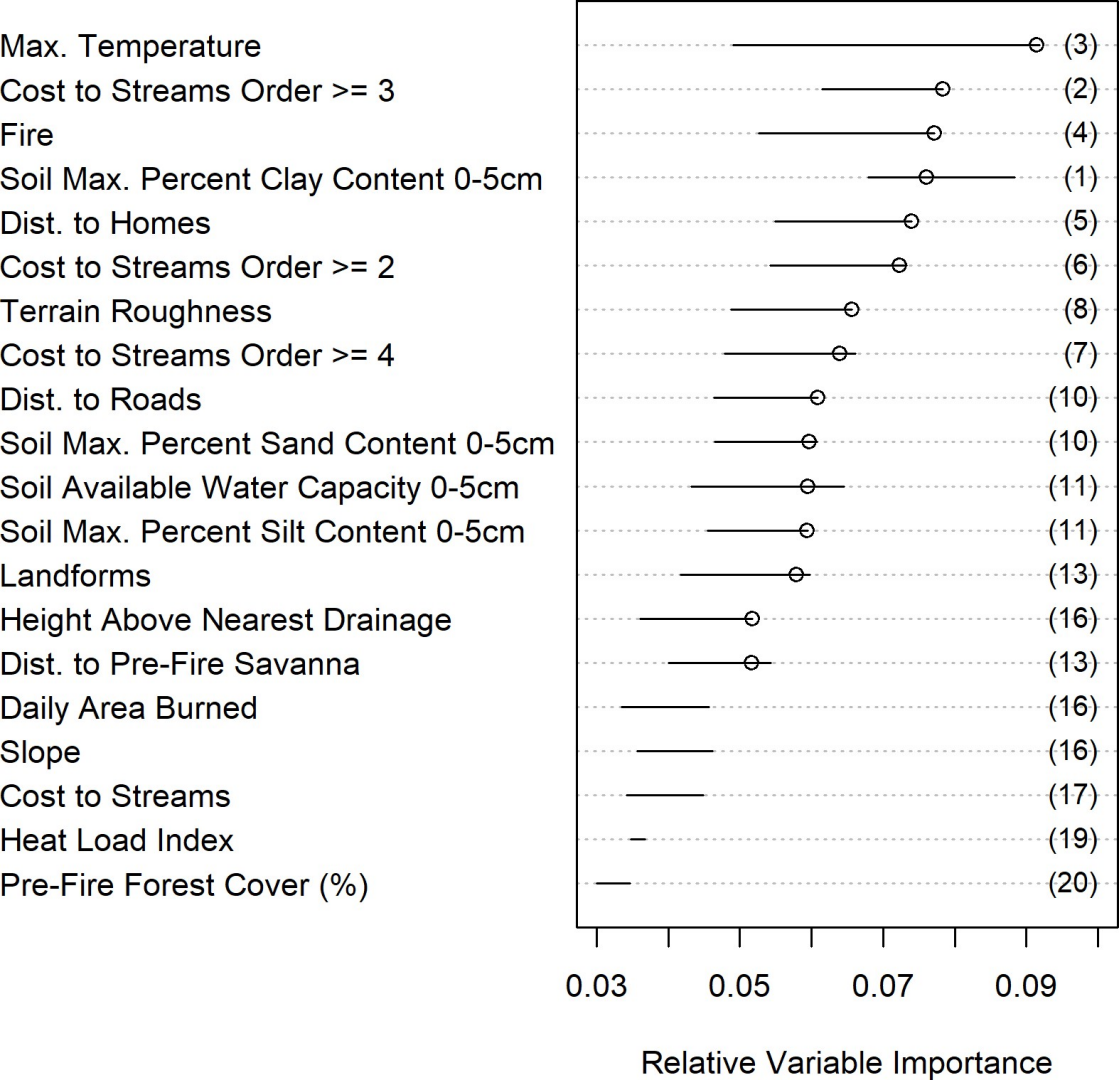
Relative variable importance in predicting Conifer Refugia. Results of Random Forest model shown in Relative Variable Importance (relativized Mean Decrease in Accuracy) for 20 variables for predicting location of Conifer Refugia within 23 wild fires that burned throughout ponderosa pine-dominated forests in the Colorado Front Range from 1996–2013. Presented variables (20 out of 25) were selected for model inclusion in at least six of the 11 model iterations. Mean decrease in accuracy is the normalized difference of the accuracy of the classification when the data for that variable are included versus when they have been randomly permutated. Values were relativized to sum to one for each model run. Higher values indicate greater importance and a higher error value when this variable is removed from the model. Dots represent the Relative Variable Importance for the primary model with the lowest Out of Bag (OOB: 23.2%) error rate, giving it an accuracy of 76.8%, and fewest selected variables (15 out of 25). Horizontal segments show the range (minimum to maximum) of Relative Variable Importance across all model iterations. Numbers in parentheses give the median ordered rank of the Relative Variable Importance across 11 model iterations.

Random forest models revealed that the most important predictors of Conifer Refugia within the 23 fire perimeters were consistently related to soil characteristics, proximity to higher order streams, maximum temperature, distance to homes, and terrain roughness (Fig 7). The 11 Random Forest models predicting Conifer Refugia and Conifer Absence included 15–22 (out of 25) predictor variables and had a mean OOB error rate of 23.7% (± 0.36%), or an average model accuracy of 76.7%. The primary model had 15 selected variables and an OOB error rate of 23.21%. The 11 Random Forest models using different subsets of the data showed consistent variable ranks and relative variable importance. The major exception was the variable Maximum Temperature, which had a wide range in variable importance across models. Soil Max. Percent Clay Content 0-5cm steadily ranked as one of most important variables, having the highest median rank across all models. Three of the top ten predictors were Cost to Streams of different orders, with higher Stream Orders ≥3 and ≥2 having the second and sixth highest median rank, respectively. Maximum Temperature was the only fire weather variable selected for modelling and ranked as the most important variable in the primary model with a median rank of third. Distance to Homes was the highest ranked anthropogenic variable with a median rank of fourth. Distance to Roads was also typically in the top ten predictors. In the primary model, Distance to Pre-Fire Savanna ranked 15^th^ in relative variable importance and Pre-Fire Forest Cover was not an important predictor of Conifer Refugia, consistent with our overlay analysis (Fig 5B).

**Fig 8:**
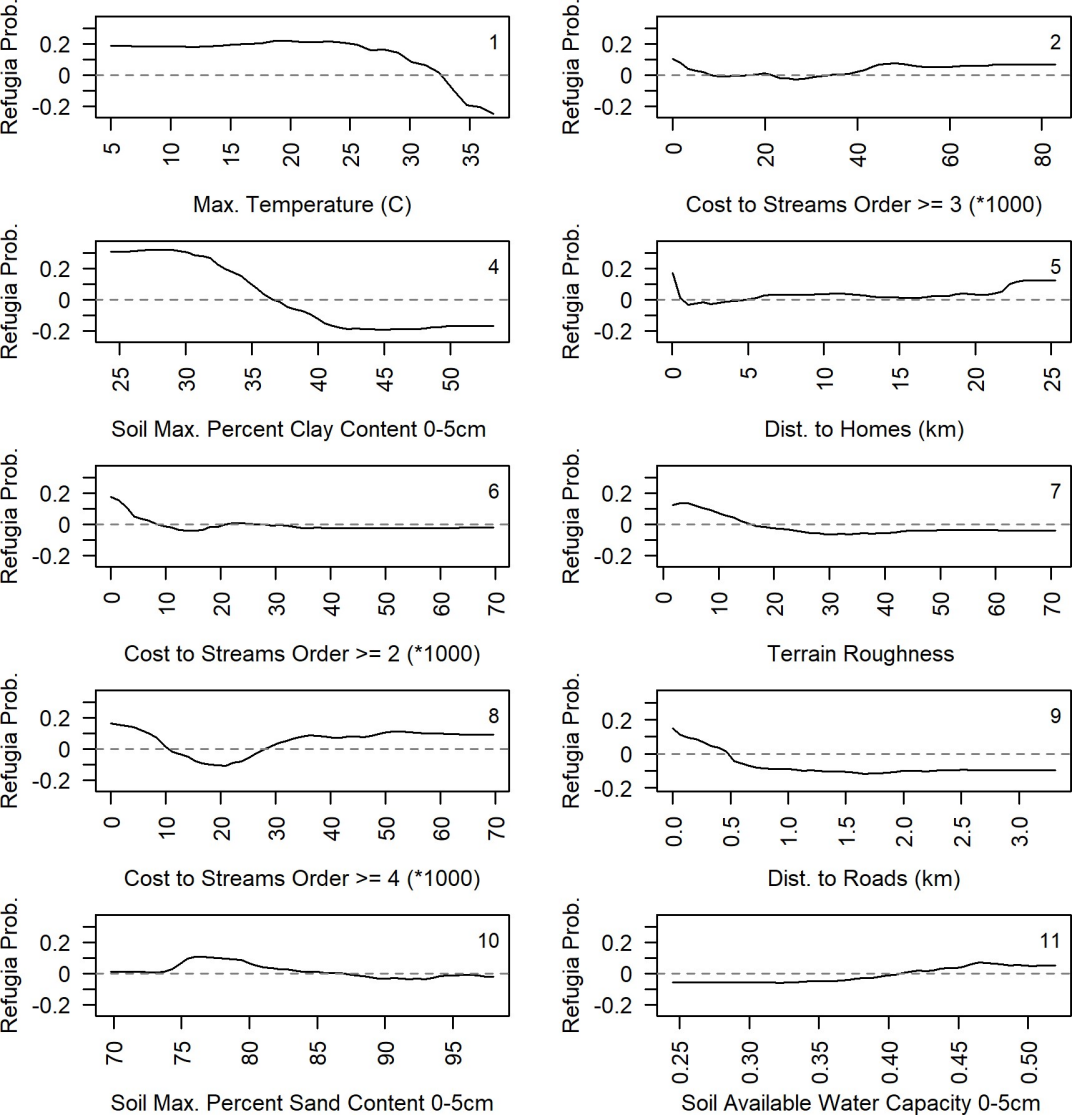
Partial-dependence plots for variables predicting Conifer Refugia. The random forest partial-dependence plots for continuous variables for the primary model with the lowest OOB and fewest selected variables (n = 15). Legend provides rank of variable importance. Partial dependence plots show the dependence of the probability of Conifer Refugia on each individual predictor variable after averaging out the effects of the other predictor variables. The y axis (Refugia Prob.) is defined as the logit probability of Conifer Refugia/2. Values for Refugia Prob. above zero indicate a higher likelihood of the presence of Conifer Refugia and value below zero indicate a higher likelihood of the absence of Conifer Refugia.

**Fig 9:**
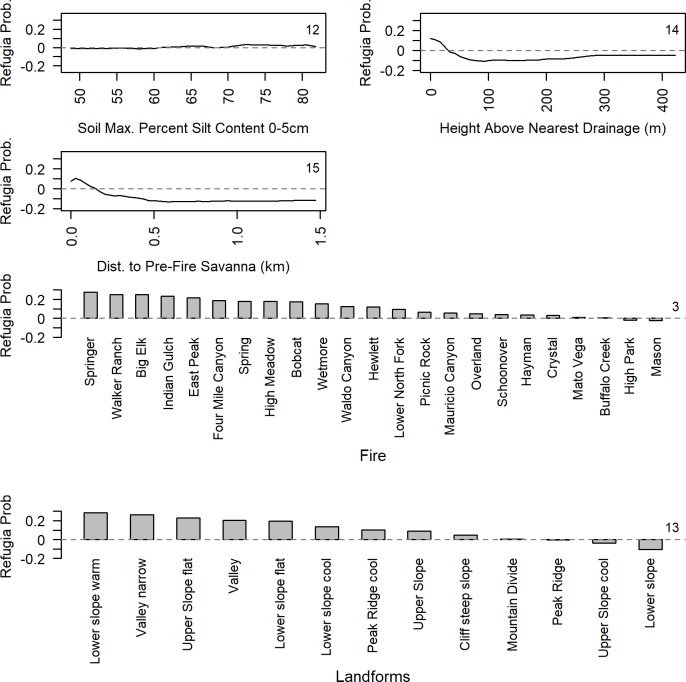
Partial-dependence plots for variables predicting Conifer Refugia. The random forest partial-dependence plots for continuous and categorical variables for the primary model with the lowest OOB and fewest selected variables (n = 15). Legend provides rank of variable importance. Partial dependence plots show the dependence of the probability of Conifer Refugia on each individual predictor variable after averaging out the effects of the other predictor variables. The y-axis (Refugia Prob.) is defined as the logit probability of Conifer Refugia/2. Values for Refugia Prob. above zero indicate a higher likelihood of the presence of Conifer Refugia and value below zero indicate a higher likelihood of the absence of Conifer Refugia.

Partial dependence plots, which show the nature of the relationship between the predictor variables and the likelihood of Conifer Refugia, indicate that lower temperatures during burning, and moister settings across the landscape increased the probability of Conifer Refugia (Fig 8 and Fig 9). Specifically, at maximum daily temperatures above ~32°C, the probability of Conifer Refugia decreased drastically. Lower Costs to Streams, which is an effective measure of proximity to a stream over sloped terrain, increased the probability of Conifer Refugia. The probability of Conifer Refugia was also inversely proportional to Max. Percent Clay Content in the 0–5 cm layer. Higher Soil Available Water Capacity, lower Soil Max. Percent Sand Content 0–5 cm, higher Soil Max. Percent Silt Content 0–5 cm, and topographic positions near drainages (Height Above Nearest Drainage <40 m) all increased the probability of Conifer Refugia. Additionally, smoother terrains and valleys, especially narrow valleys, and flat sloped landforms coincided with a higher probability of Conifer Refugia. The unique characteristics of each fire also played an important role in predicting Conifer Refugia, with certain fires having higher probability of Conifer Refugia. We provided visual examples of the variation in Conifer Refugia after different fires across three distinct landscapes (S1 Fig).

## Discussion

Utilizing fine-resolution (1m) aerial imagery to map post-fire Conifer Refugia within 23 fires that burned ponderosa pine-dominated forests of the CFR from 1996–2013 allowed us to thoroughly examine the spatial characteristics and predictability of Conifer Refugia in this region. Our study of post-fire surviving trees found that over one-third of the area burned in the CFR resulted in large contiguous patches without surviving trees at distances greater than the common dispersal distance of ponderosa pine seeds, presenting a potential forest recovery challenge. We generally found that abiotic and topographic landscape variables, many indicative of moister microsites, were the most important predictors of conifer refugia, except under high maximum temperatures and the most extreme fire weather, in which case conifers were generally less likely to survive. The lack of a strong relationship between pre-fire forest cover and post-fire Conifer Refugia may have strong implications for forest management aimed at increasing the resilience of ponderosa pine to wildfire through tree thinning. We recommend prioritizing RdNBR over TBSC as a management tool to more accurately identify post-fire conifer refugia, evaluate patch metrics of surviving post-fire conifers, and plan for post-fire recovery.

### Spatial characteristics of Conifer Refugia and Conifer Absence

Post-fire forested refugia are important disturbance legacies providing seed sources and microsites for recovery of ponderosa pine forests [21,22,24,75]. Yet, our study within 23 wildfires along the CFR from 1996–2013 found that 58% of the burned area did not contain surviving post-fire conifers. Thirty-eight percent of the burned area was greater than 50m from a surviving conifer, may have inadequate regeneration of ponderosa pine due to limited seed source, and may transition to non-forest or shrubland, or require long periods to recover to forests [14,24,28,76,77]. These burned areas were largely forested prior to burning (95% presence of forest cover), highlighting a potential large reduction in forest cover in these areas if regeneration is not adequate. The high abundance of numerous small patches of Conifer Refugia are ecologically significant and reduced the total area distant from seed sources, except within the largest high-severity patches. These small patches of refugia can provide a disproportionate amount of regeneration in burned ponderosa pine forest compared to larger patches of surviving trees [51]. Our fine-scale spatial evaluation confirms for the CFR a lack of small forested fire refugia in the interior of large high-severity patch centers, usually documented at a coarser 30-m resolution [4,5,12,13,19].

Evaluating both the spatial and temporal trends in Conifer Refugia proved critical to understanding the potential effectiveness of these legacies in promoting post-fire recovery. We found a consistent proportion of Conifer Refugia across time in the CFR, similar to findings during the last two decades in the northern Rockies [34]. Although post-fire recovery in the northern Rockies might not be impacted by recent increases in fire severity due to the consistent proportion of fire refugia within wildfires [34], we found that in the CFR (regardless of the lack of trend in proportion of Conifer Refugia), the spatial arrangement of forested fire refugia is a key component to evaluating the potential resilience to fire for ponderosa pine. In our study, Conifer Refugia occupied 42% of the total area within burn perimeters. Although we found an abundance of ecologically important small patches of Conifer Refugia within fire perimeters, each fire contained at least one large patch of contiguous treeless area. The size of the largest treeless patch increased directly with fire size and is predicted to be approximately a third of the fire size. Total area of forested fire refugia may underestimate the management challenge of large patches of post-fire tree mortality, especially in forests dominated by obligate-seeding species with shorter seed-dispersal distances.

In the Southern Rockies, from 1984 to 2006 there was a trend of increasing area burned [78], which may contribute to trends in high-severity fire in the region [79] and increasingly large high-severity post-fire patches without surviving trees. This study contributes to a growing body of evidence that patch size of high-severity fire scales with fire size across western ecoregions [4,5]. This relationship may have greater ecological consequences for non-sprouting woody species with larger seeds that rely partly on animal dispersion, such as ponderosa pine [80]. Numerous studies have documented the importance of a close distance to seed source (≥50m) for more abundant ponderosa pine regeneration [22–24,50,26,75]. Concerns about limited post-fire regeneration will further magnify as fire size is expected to increase with current warming patterns [3,4]. Therefore, although larger fires seem to provide conifer refugia in similar proportion to smaller fires, larger fires are more likely to contain extensive, treeless areas in ponderosa pine dominated forests in this region, creating an important current and future management challenge.

Limited long-distance dispersal into high-severity patches has been documented [50]. Such dispersal is likely mediated by animals, and may have been facilitated by sprouting woody trees (gambel oak) within the patches [50]. Sprouting woody trees may attract and create habitat for seed dispersers like Clark’s nutcracker, scrub jay, and pinyon jay [50]. In the absence of woody shrubs, there is reduced regeneration in interior high-severity patch locations [50]. In the current study, the maximum distance from a potential conifer seed source increased on average by over 400 m across all fires and was over 1000 m in three fires, indicating the size of these large high-severity patches. It is currently unknown if long distance dispersal of ponderosa pine is reaching the interiors of the largest treeless patches (even in areas with sprouting aspen regeneration), but the current evidence suggests that regeneration in these areas would be lacking or extremely scarce [22,24].

### Predictability of Conifer Refugia using MTBS burn severity metrics

The use of high-resolution imagery allowed us to accurately map conifers at very low densities and thoroughly evaluate the ability of coarser scale burn severity indices to detect these conifer refugia. We found that post-fire conifer refugia are not consistently detected by MTBS TBSC and that these classes generally under-estimated conifer loss in the ponderosa pine cover type in our region. Although thematic burn severity metrics typically capture changes in all organic matter above and below ground, such as soil, understory vegetation, and canopy mortality [81], we focused on overstory conifer forest mortality. While TBSC generally predicted complete conifer mortality in the High burn severity class, Low and Moderate burn severity classes also had a high portion of area without surviving conifers. Loss of seed trees in these post-fire environments may be more significant than previously inferred from TBSC. Studies using TBSC to generate patch metrics of areas lacking conifer tree cover may greatly underestimate the extent of canopy mortality and overestimate the potential for recovery in recent fires (e.g., [13]). The ability of thematic burn severity classes to detect conifer mortality was influenced by tree canopy cover. Lower tree covers were more likely to be classified as Low and Moderate burn severity even though half of these areas suffered 100% canopy mortality. Studies using TBSC provided by MTBS or other dNBR-based metrics (as opposed to RdNBR) to evaluate predictors of burn severity may overestimate the role of vegetation cover and underestimate the role of other factors in contributing to 100% canopy mortality (e.g., [82]). Our results further corroborate research documenting the limitations of MTBS TBSC to accurately account for ecological metrics in burn severity [83].

RdNBR provided a more robust method for detecting post-fire surviving conifers and we recommend its use in post-fire recovery planning in ponderosa pine-dominated forests. RdNBR is more consistent in classifying burn severity in areas with low forest cover and has identified fire refugia in other regions [34,78]. Our analysis corroborates similar recent research in ponderosa pine and dry-mixed conifer forests of the western US demonstrating the high predictive power of RdNBR and fine scale imagery to detect fire refugia [84]. Overall, our findings support the use of RdNBR products to identify presence of post-fire conifer refugia in ponderosa pine dominated forests.

### Predictability of Conifer Refugia using landscape variables: Weather, anthropogenic, biotic, abiotic, and fire factors

Conifer refugia were least likely to survive wildfires occurring under the highest temperatures, highest wind speeds, and driest conditions. Higher daily maximum temperature was the highest ranked variable in the primary random forest model predicting higher tree mortality. Our findings generally corroborate other studies showing the decreased importance of topographic variables in predicting fire severity and fire refugia under warmer, drier, and more extreme burning conditions [4,30,31,78]. In our study, under more moderate fire weather, the total area of surviving conifers was approximately equal to the area lacking surviving trees. However, more than half of the area within fire perimeters (73%) burned under extreme fire weather which explains the higher total proportion of treeless post-fire areas. Historically, both climate and topography influenced low, mixed, and high fire severity patterns in the region [6,9,85]. Our study suggests a decreased likelihood of forested refugia and low-severity fire under extreme fire weather in current forest conditions. With increased warming, fire weather and fuel aridity are predicted to increase fire severity across ecoregions in the western US [86]. The lengthening of the fire season across the West due to earlier snow melt and warmer temperatures also increases the chance of extreme burning conditions [87]. The timing of Colorado’s snow melt has already shifted 1–4 weeks earlier since 2000 and the Palmer Drought Severity Index (PDSI) also shows a trend toward more severe drought from 1983 to 2012 [88]. According to our study, these climate trends and predictions will favor extreme burning days with a low portion of surviving trees on those days.

Our hypothesis that abiotic factors have an influential role in providing post-fire refugia across the CFR was generally supported. The most important landscape variables in predicting Conifer Refugia were related to soil characteristics, proximity to higher order streams, less rugged terrain, valley landforms, and lower elevation in drainages. The partial dependence plots of the Random Forest model suggest that post-fire conifer survivorship in ponderosa pine dominated forests is generally predicted in moister or less steep sites. Conifer survivorship in locations near the floodplain or watershed drainage is most likely influenced by soil properties, microclimates, and topography. Wetlands, stream confluences, riparian areas, valley bottoms and cold air drainages can have decreased fire severities [29,89–92]. As we found in our study, higher stream orders can also have lower fire severity [93].

Soil properties in the top five centimeters heavily influenced the presence of Conifer Refugia. Quantitative studies of ponderosa pine densities and soil texture in the Southwest have shown that soils with a high clay content are less favorable for ponderosa pine and more favorable for competing grasses [32,94,95]. Additionally, post-fire ponderosa pine regeneration can be more dense in areas with lower clay content [95]. These relationships are consistent with qualitative observations in the CFR that ponderosa pine is less abundant on soils with high clay content such as the lower slopes and valley bottom sites in the lower montane zone [45]. On finely-textured soils grasses may competitively exclude trees whereas deeper moisture infiltration on coarsely textured soils may be more favorable to trees. Strong associations between understory vegetation and historical fire regimes have been documented in the CFR and may also be influenced by soil properties [96]. We found that lower clay content in the top five centimeters increased probability of post-fire conifer survivorship which hypothetically could reflect better tree survival on rocky sites lacking continuous surface fuels (S1A Fig). The soil layers used in our analysis are predictions based on the Soil Survey Geographic (SSURGO) database and a suite of topographic indices [68], which may add to their importance in predicting conifer refugia. Although the causal relationship between low clay content and increased fire refugia is beyond the scope of our analysis, our study suggests the need for forest management to consider factors including cooler, moister topographies and soil properties that promote forested fire refugia as burning conditions become hotter and drier [94].

Anthropogenic influences also promoted conifer survivorship within our study area. Both proximate distance to homes and distance to roads were consistently ranked in the top ten predictors of Conifer Refugia. Roads may serve as natural fuel breaks or be used to strategically locate active fire suppression efforts, both promoting survivorship of trees in the area. Homes are also actively protected from oncoming wildfires with wildfire suppression tactics and numerous private land owners in the CFR treat fuels adjacent to their homes [39,97]. Tree survivorship near homes may be the combined result of active fire suppression tactics during the fire and private home mitigation. Fuel treatments near communities can be actively used to engage in fire suppression tactics near neighborhoods [98] and may also act to promote conifer refugia near homes. Larger-scale forest fuel treatments may also provide long-term carbon storage benefits primarily in dry forests experiencing frequent fire (1–2% probability of wildfire per year) [99,100]. The degree to which forest fuel treatments, thinning and prescribed burning, provide both short-term and longer-term conditions conducive to post-fire conifer refugia in the face of growing fire size and extreme fire weather is highly dependent on fire likelihood, and is an area of critical future research. While our analysis identified Conifer Refugia in single fire events, distinguishing between ephemeral and persistent fire refugia may require additional fire modelling and could greatly inform prioritized locations of forest fuel mitigation treatments.

Biotic variables, distance to savanna and pre-fire forest cover, were not strong predictors of Conifer Refugia. Overlays showed that forest cover under 60% was only slightly more likely to survive these wildfires and half of the area with forest cover between 20% and 50% had no surviving conifers. Although forest cover overall had a very weak relationship with tree survivorship, the proximate distance to savannas (open areas and forest cover ≤ 20%) had a stronger influence. While elevation is historically the single most important predictor of low-severity fire within the CFR [54], forests closer to grasslands also tend to have historical low fire severity [101]. Open meadows with lower fire severity and higher conifer tree survivorship may be an important component to post-fire refugia and recovery. We did not account for the strong influence of fine fuels, understory vegetation, and fuel continuity, which may limit our evaluation of forest cover in spreading and carrying wildfire within denser forest covers.

The importance and decline of small meadows in ponderosa pine has been documented in Colorado [102]. Between 1938 and 2015 fire-excluded areas with a historic low-severity fire regime in the lower montane of the CFR experienced an almost 16% net increase in forest cover and fire-excluded mixed-severity stands showed an almost 12% net increase [58]. While mixed- and high-severity fire has been an integral part of ponderosa pine forests in the Southern Rockies in higher elevations [6,7,54], the presence of small patches of surviving trees within and bordering savannas may have been a critical aspect of ecosystem recovery, particularly at lower elevations with low-severity fire regimes [11,53,59]. Savannas may act similarly to roads and slow the fire front so that trees survive both in the meadow and in surrounding densely forested area. The extent of these open forest areas has declined, potentially creating greater woody fuel continuity and larger high-severity patches documented in this study.

In addition to the decline in area of meadows along the CFR, there appears to be a surprising lack of resistance to recent fire in the remaining savannas. Nearly half of the canopy cover of the savannas within the fire perimeters did not survive. The maintenance and protection of large, old trees is important for both ecosystem services and carbon storage [103]. The high mortality in more open savannas, thought to foster lower fire severities and older trees, may be indicative of lowered forest resilience in this forest type. Historical reconstructions in the southern CFR found that 30% of all stands with minimal human disturbance had trees over 400 years old [104]. However, the 2002 Hayman fire (the largest fire in our study) killed the vast majority of old trees in its path [105]. The high percentage of low forest covers that suffered 100% mortality across all fires may be indicative of a loss of formerly more persistent refugia across the study area and requires field verification. Forest management may prioritize prescribed burning of existing savannas and meadows as a crucial first step in potentially increasing tree survivorship in savannas and in the surrounding denser forests [106]. A better understanding of drivers of more persistent meadows and associated soil properties in the CFR could also help guide management in locating areas for meadow restoration and may drive more innovative management strategies for fuel treatments, such as small meadow restoration for conifer refugia [102].

Our analysis highlights specific opportunities regarding forest management to enhance forest resilience in the face of increased wildfires under a warming climate [107–109]: 1) utilize the abiotic predictors analyzed in the current study to create predictive maps of probabilities of forest refugia under a range of fire weather scenarios; 2) consider prioritizing forest fuel mitigation treatments in habitats of greater predicted tree survivorship, particularly in areas where prescribed burning is less likely due to safety (e.g. WUI) and smoke concerns; and 3) increase the focus on treatments in savannas, where tree removal would be minimal and prescribed fire is more feasible so that tree survivorship is enhanced to assure seed sources for recovery from future large fires.

## Conclusion

Although 42% of the total burned area contained surviving post-fire conifers in our study of 23 fires, mechanisms of resilience of ponderosa pine systems to wildfire may be declining along the CFR. We highlight four general findings that underscore the potential declining resilience of ponderosa pine forests in Colorado with a continued trend of increasing area burned under a warming climate: 1) large fires are expected to have large patches approximately a third the size of the fire area that lack surviving conifers and therefore seed sources for regeneration; 2) wetter areas and topographic low points generally have a higher likelihood of conifer presence following wildfires; 3) forest cover was not a strong predictor of conifers surviving wildfire; and 4) conifers are less likely to survive under extreme fire weather and burning conditions. Research in the CFR and elsewhere has reported decreased ponderosa pine regeneration in response to warmer drier climates following wildfires. These same climate conditions are promoting large wildfires with extensive treeless patches, further compromising post-fire resilience to wildfire. For ponderosa pine forests in the CFR, the combination of larger fires and warmer-drier burning conditions poses a potentially great threat of forest loss with a lack of post-fire recovery due to lack of seed sources alone. Even patches of the lowest tree cover in ponderosa pine forests suffered high mortality in recent fires along the CFR. Our results suggest numerous management implications including: the preference of RdNBR over TBSC to evaluate post-fire surviving seed sources, the importance of including soil and topographic variables into management planning to promote post-fire conifer refugia, the increased use of prescribed fire in existing meadows and woodlands to potentially increase the survivorship of conifer trees, and a reprioritization that may include wide-scale planting of seedlings for this forest type in areas lacking seed sources but at sites where they are more likely to survive future fires.

## Supporting information

S1 Table Existing Vegetation Types by individual fires. Percent cover of forest and woodlands in the 2001 LANDFIRE Existing Vegetation Types within 23 fires that burned ponderosa pine-dominated forests along Colorado’s Front Range 1996–2013.
(DOCX)

S2 Table Existing Vegetation Types for all fires. Total percent cover and area of forest and woodlands in the 2001 LANDFIRE Existing Vegetation Types across 23 fires that burned ponderosa pine-dominated forests along Colorado’s Front Range 1996–2013.
(DOCX)

S3 Table Summary of 25 predictor variables. Importance and data sources for landscape variables generated for the random forest model meant to classify Conifer Refugia and Conifer Absence. Selected variables in more than six of 11 Random forest models are indicated by *. An expected positive relationship between the variable and Conifer Refugia is denoted with (+) and an expected negative relationship with (-). All variables are at 30-m resolution.
(DOCX)

S4 Table Distance to potential seed source by fire. Maximum, mean, and standard deviation of distance to potential pre- and post-fire seed source for the 23 fires that burned ponderosa pine-dominated forests along Colorado’s Front Range 1996–2013.
(DOCX)

S1 Fig Examples of Conifer Refugia. Three examples of Conifer Refugia within 23 fires that burned ponderosa pine-dominated forests along Colorado’s Front Range 1996–2013 as shown by 2015 NAIP aerial imagery.
(TIF)

S1 Text Methods for Daily Fire Weather and Pre-Fire Forest Cover. (DOCX)
