# Supplementary material for: Still standing: Recent patterns of post-fire conifer refugia in ponderosa pine-dominated forests of the Colorado Front Range
Source: PLoS One. 2020 Jan 15;15(1):e0226926. doi: 10.1371/journal.pone.0226926 (PMC6961861; doi:10.1371/journal.pone.0226926)
Supplement: S1 Table — Percent cover of forest and woodlands in the 2001 LANDFIRE Existing Vegetation Types within 23 fires that burned ponderosa pine-dominated forests along Colorado’s Front Range 1996–2013. (DOCX) [file pone.0226926.s001.docx]

|  | Landfire Existing Vegetation Types | | | | | | | | |  |  |
| --- | --- | --- | --- | --- | --- | --- | --- | --- | --- | --- | --- |
|  | Southern Rocky Mountain | | | | Inter Mountain Basin | Rocky Mountain | | Colorado Plateau | Other |  |  |
| Fire (Year Fire Name) | Ponderosa Pine Wood-land | Dry Mesic Montane Mixed Conifer Forest and Wood-land | Mesic Montane Mixed Conifer Forest and Wood-land | Pinyon Juniper Wood-land | Aspen Mixed Conifer Forest and Woodland | Lodge-pole Pine Forest | Aspen Forest and Wood-land | Pinyon Juniper Wood-land | Forest and Wood-land | Total Forest Cover (%) | Fire Area (ha) |
| 1996 Buffalo Creek | 22.6 | 69.8 | 0.7 | 0.3 | 0.5 | 0 | 0.1 | 0.4 | 0.1 | 94.5 | 3966 |
| 2000 Bobcat | 70 | 22.2 | 0.1 | 0.5 | 2.3 | 1.3 | 1.1 | 0.1 | 0.2 | 97.8 | 3669 |
| 2000 Eldorado | 52.9 | 35.4 | 2.1 | 4.6 | 0.7 | 0 | 0.2 | 0 | 0.4 | 96.3 | 398 |
| 2000 High Meadow | 17.3 | 66.4 | 11 | 0.4 | 1 | 0.1 | 0.1 | 1 | 0.1 | 97.4 | 3859 |
| 2002 Big Elk | 24.8 | 24.6 | 4.4 | 0 | 7.5 | 32.9 | 1.4 | 0 | 0.3 | 95.9 | 1743 |
| 2002 Hayman | 15.4 | 61.5 | 5.7 | 0 | 8.9 | 0.3 | 2.1 | 0.3 | 0.6 | 94.8 | 52167 |
| 2002 Schoon-over | 45.2 | 49.3 | 0 | 0.5 | 0.1 | 0 | 0.2 | 1.3 | 0.3 | 96.9 | 1136 |
| 2002 Spring | 20 | 51.9 | 5.7 | 0 | 6.1 | 0 | 3.6 | 0.4 | 3.9 | 91.6 | 9729 |
| 2003 Overland | 51.3 | 34.4 | 1.6 | 0.2 | 4 | 1.7 | 1.5 | 0 | 0.2 | 94.9 | 1292 |
| 2004 Picnic Rock | 28 | 11.2 | 0 | 0.4 | 8.8 | 0 | 0.3 | 1.8 | 0.1 | 50.6 | 3264 |
| 2005 Mason | 18.7 | 57.7 | 0.2 | 0.1 | 0.6 | 0 | 0.1 | 13.2 | 0 | 90.6 | 4194 |
| 2006 Mato Vega | 8.9 | 12.1 | 15.9 | 0 | 33.7 | 0.1 | 7.8 | 10.6 | 0.7 | 89.8 | 5312 |
| 2006 Mauricio Canyon | 49.4 | 7.9 | 8.3 | 0.2 | 1.2 | 0 | 10.6 | 3.2 | 0 | 80.8 | 1770 |
| 2010 Four Mile Canyon | 54 | 29.2 | 3.2 | 1.2 | 3.1 | 0.4 | 1.5 | 0 | 0 | 92.6 | 2347 |
| 2011 Crystal | 49.8 | 20.4 | 0.3 | 1.2 | 4 | 3.2 | 1.9 | 0 | 0.1 | 80.9 | 1120 |
| 2011 Indian Gulch | 22.6 | 27.2 | 5.1 | 5.3 | 5.4 | 0 | 0.7 | 0.2 | 0 | 66.5 | 651 |
| 2012 Hewlett | 48.4 | 27.3 | 0.2 | 2.4 | 4.2 | 0 | 0.3 | 0.9 | 0.1 | 83.8 | 2069 |
| 2012 High Park | 37.4 | 28.4 | 0.6 | 0.9 | 3.4 | 11.8 | 3 | 0.2 | 0.6 | 86.3 | 34482 |
| 2012 Lower North Fork | 8.1 | 51.5 | 16.2 | 1.7 | 5.3 | 0 | 4.4 | 0.1 | 0.3 | 87.6 | 1374 |
| 2012 Springer | 45.4 | 5.8 | 1.8 | 0 | 35.8 | 0 | 1.9 | 0.6 | 0.1 | 91.4 | 663 |
| 2012 Waldo Canyon | 41.7 | 27.9 | 9.2 | 0.6 | 6.5 | 0.3 | 2.1 | 1.1 | 2.9 | 92.3 | 8086 |
| 2012 Wetmore | 8.5 | 16.9 | 0 | 19 | 1.3 | 0 | 0.1 | 38.8 | 2.1 | 86.7 | 846 |
| 2013 East Peak | 32.3 | 7.7 | 27.9 | 0 | 13.2 | 0 | 7.9 | 0.9 | 1.6 | 91.5 | 4081 |
| Average | 33.6 | 32.5 | 5.2 | 1.7 | 6.9 | 2.3 | 2.3 | 3.3 | 0.2 | 88.3 |  |
| (+- SD) | (17.1) | (19.2) | (6.8) | (3.9) | (9.1) | (7.0) | (2.7) | (8.3) | (0.3) | (10.9) |  |
