# Supplementary material for: Still standing: Recent patterns of post-fire conifer refugia in ponderosa pine-dominated forests of the Colorado Front Range
Source: PLoS One. 2020 Jan 15;15(1):e0226926. doi: 10.1371/journal.pone.0226926 (PMC6961861; doi:10.1371/journal.pone.0226926)
Supplement: S2 Table — Total percent cover and area of forest and woodlands in the 2001 LANDFIRE Existing Vegetation Types across 23 fires that burned ponderosa pine-dominated forests along Colorado’s Front Range 1996–2013. (DOCX) [file pone.0226926.s002.docx]

| 2001 Landfire Existing Vegetation Type | Landfire Value | Percent | Area (ha) |
| --- | --- | --- | --- |
| Southern Rocky Mountain Dry-Mesic Montane Mixed Conifer Forest and Woodland | 2051 | 42.7 | 63198 |
| Southern Rocky Mountain Ponderosa Pine Woodland | 2054 | 27.0 | 39945 |
| Inter-Mountain Basins Aspen-Mixed Conifer Forest and Woodland | 2061 | 7.1 | 10466 |
| Southern Rocky Mountain Mesic Montane Mixed Conifer Forest and Woodland | 2052 | 5.1 | 7546 |
| Rocky Mountain Lodgepole Pine Forest | 2050 | 3.4 | 4960 |
| Rocky Mountain Aspen Forest and Woodland | 2011 | 2.6 | 3816 |
| Colorado Plateau Pinyon-Juniper Woodland | 2016 | 1.4 | 2066 |
| Quercus gambelii Shrubland Alliance | 2217 | 0.8 | 1126 |
| Southern Rocky Mountain Pinyon-Juniper Woodland | 2059 | 0.5 | 786 |
| Rocky Mountain Subalpine Dry-Mesic Spruce-Fir Forest and Woodland | 2055 | 0.4 | 597 |
| Rocky Mountain Gambel Oak-Mixed Montane Shrubland | 2107 | 0.2 | 269 |
| Rocky Mountain Subalpine-Montane Limber-Bristlecone Pine Woodland | 2057 | 0.1 | 132 |
| Southern Rocky Mountain Ponderosa Pine Savanna | 2117 | 0.0 | 37 |
| Total |  | 91.2 | 134946 |
