# Supplementary material for: Still standing: Recent patterns of post-fire conifer refugia in ponderosa pine-dominated forests of the Colorado Front Range
Source: PLoS One. 2020 Jan 15;15(1):e0226926. doi: 10.1371/journal.pone.0226926 (PMC6961861; doi:10.1371/journal.pone.0226926)
Supplement: S3 Table — Importance and data sources for landscape variables generated for the random forest model meant to classify Conifer Refugia and Conifer Absence. Selected variables in more than six of 11 Random forest models are indicated by *. An expected positive relationship between the variable and Conifer Refugia is denoted with (+) and an expected negative relationship with (-). All variables are at 30-m resolution. (DOCX) [file pone.0226926.s003.docx]

| Variable Name and Expected Relationship with Conifer Refugia | Importance for Conifer Refugia and Data Source |
| --- | --- |
| Fire weather variables | |
| 1. Maximum Temperature* (-) | In ponderosa pine forests, high severity fire occurs in large patches on extreme fire weather days [1–3]. Data: Closest Remote Automatic Weather Station (RAWS) daily weather summary for fire weather [4], Burn date established by MODIS Burned Area Products [5] and GeoMAC historic fire perimeters [6], and Fire danger Rating Data: USFS Wildland Fire Assessment System [7]. |
| 2. Maximum Wind Speed (-) |  |
| 3. Minimum Relative Humidity (-) |  |
| 4. Fire Danger Rating (-) |  |
| Anthropogenic influence variables | |
| 5. Distance to Homes* (+ or -) | Firefighting actively protects homes during wildfires, which may increase the likelihood of trees near homes. Home mitigation may also serve to protect trees. However, home ignition is based on building materials and vegetation close to home and may be independent of fire severity [8]. Data: Point home locations along Front Range of Colorado [9]. Home locations in counties not included in Caggiano et al (2016) were hand digitized with NAIP 2015 1m imagery. |
| 6. Distance to Roads* (+) | Roads may act to slow fire spread or fire fighters may use roads in suppression tactics, such as back burning. Data: County Tiger Road lines [10]. |
| Biotic variables | |
| 7. Pre-Fire Forest Cover* (+ or -) | Forest density in ponderosa pine has increased relative to historical levels in some regions and is thought to increase fire severity [11]. Reconstructions in the CFR found historic low severity fire at lower elevations, where ponderosa pine typically forms low density patches based on topography and moisture [12]. Lower-severity fire could likely lead to higher refugia presence. At higher elevations where ponderosa pine typically forms denser stands, historic reconstructions found a dominance of mixed and high severity fire [13], potentially predicting lower refugia presence. Data: 2001 NLCD Percent Tree Canopy, based off Landsat satellite classification [14], 1999 1m DOQQ black and white aerial images and 1994 BW aerial images [15]. |
| 8. Pre-Fire Distance to Savanna* (-) | Ponderosa pine forests near grasslands had higher fire frequencies and lower fire severities historically [16]. Data: Pre-fire Forest Cover variable. |
| Abiotic variables | |
| 9. Cost to Streams Order >= 4* (-) | Wetlands, stream confluences, higher stream orders, and cold air drainages can all have reduced fire severity [17–20]. Data: National Hydrography Database Plus V2 [21], HAND Downloaded from Google Earth Engine [22]. |
| 10. Cost to Streams Order >= 3* (-) |  |
| 11. Cost to Streams Order >= 2* (-) |  |
| 12. Cost to Streams (-) |  |
| 13. Height Above the Nearest Drainage* (HAND) (-) |  |
| 14. Compound Topographic Index (CTI) (+) | Burned areas with higher wetness can have increased probability of forested refugia under all burning weather conditions [2]. Data: USGS DEM [23]. |
| 15. Terrain Roughness* (+ or -) | More rugged terrain may increase fire spread or lower predictability of refugia [1,2]. Rugged terrain can also support lower fire severity, depending on region of analysis [24]. Data: USGS DEM [23]. |
| 16. Heat Load Index* (HLI) (+) | Warm, south facing slopes can be likely to burn at high severity [25]. Data: USGS DEM [23]. |
| 17. Aspect* (+) | Lower severity fire in ponderosa pine forests can be found on south facing slopes [24]. Likewise, fire refugia can have reduced likelihood on northern aspects [26]. Data: Landfire aspect [23]. |
| 18. Slope* (+ or -) | Steep slopes can have higher likelihood of fire refugia under all weather conditions [2]. Flatter slopes also can have increased fire refugia under moderate fire weather [2,26]. Data: Landfire slope [23]. |
| 19. Landforms* (+ or -) | Valley bottoms and upper headwalls can have a higher proportion of refugia [26]. Data: [27]. |
| 20. Soil Max. Percent Clay Content 0-5cm* (-) | Historic ponderosa pine densities can be predicted by soil properties in the top 0-15 cm, such as percent silt and available water capacity, in the southwest [29]. Both high clay and high sand soils can support lower density historic stands, which supported lower fire severities [28]. Soil properties may also influence understory vegetation, which has been related to historic fire severity in the CFR [29]. Higher silt content and higher available water capacity may also indicate areas close to riparian corridors, which can experience lower fire severity [18]. Data: Polaris (A 30-meter probabilistic soil series map of the contiguous United States [30]. |
| 21. Soil Max. Percent Silt Content 0-5cm* (+) |  |
| 22. Soil Available Water Capacity 0-5cm* (+) |  |
| 23. Soil Max. Percent Sand Content 0-5cm* (-) |  |
| Fire variables | |
| 24. Fire* | Fires can create their own weather and be driven by random shifts in winds, resulting in varying patterns of fire refugia unique to that fire [31]. |
| 25. Daily Area Burned | Patch size may explain spatial autocorrelation [32]. |

**References**

1. Holsinger L, Parks SA, Miller C. Weather, fuels, and topography impede wildland fire spread in western US landscapes. Forest Ecology and Management. 2016;380: 59–69. doi:10.1016/j.foreco.2016.08.035

2. Krawchuk MA, Haire SL, Coop J, Parisien M-A, Whitman E, Chong G, et al. Topographic and fire weather controls of fire refugia in forested ecosystems of northwestern North America. Ecosphere. 2016;7: e01632. doi:10.1002/ecs2.1632

3. Odion DC, Hanson CT, Arsenault A, Baker WL, DellaSala DA, Hutto RL, et al. Examining Historical and Current Mixed-Severity Fire Regimes in Ponderosa Pine and Mixed-Conifer Forests of Western North America. PLOS ONE. 2014;9: e87852. doi:10.1371/journal.pone.0087852

4. RAWS USA Climate Archive State Selection Map. [cited 19 Jul 2019]. Available: https://raws.dri.edu/

5. Roy DP, Boschetti L, Justice CO, Ju J. The collection 5 MODIS burned area product — Global evaluation by comparison with the MODIS active fire product. Remote Sensing of Environment. 2008;112: 3690–3707. doi:10.1016/j.rse.2008.05.013

6. GeoMAC Wildfire Application. [cited 19 Jul 2019]. Available: https://www.geomac.gov/

7. Fire Danger Rating. [cited 19 Jul 2019]. Available: https://www.wfas.net/index.php/fire-danger-rating-fire-potential--danger-32

8. Calkin DE, Cohen JD, Finney MA, Thompson MP. How risk management can prevent future wildfire disasters in the wildland-urban interface. PNAS. 2014;111: 746–751. doi:10.1073/pnas.1315088111

9. Caggiano MD, Tinkham WT, Hoffman C, Cheng AS, Hawbaker TJ. High resolution mapping of development in the wildland-urban interface using object based image extraction. Heliyon. 2016;2: e00174. doi:10.1016/j.heliyon.2016.e00174

10. Bureau UC. TIGER/Line Geodatabases. [cited 19 Jul 2019]. Available: https://www.census.gov/geographies/mapping-files/time-series/geo/tiger-geodatabase-file.html

11. Fulé PZ, Covington WW, Moore MM. Determining reference conditions for ecosystem management of southwestern ponderosa pine forests. Ecological Applications. 1997;7: 895–908. doi:10.1890/1051-0761(1997)007[0895:DRCFEM]2.0.CO;2

12. Sherriff RL, Veblen TT. A Spatially-Explicit Reconstruction of Historical Fire Occurrence in the Ponderosa Pine Zone of the Colorado Front Range. Ecosystems. 2007;10: 311–323. doi:10.1007/s10021-007-9022-2

13. Schoennagel T, Sherriff RL, Veblen TT. Fire history and tree recruitment in the Colorado Front Range upper montane zone: implications for forest restoration. Ecological Applications. 2011;21: 2210–2222. doi:10.1890/10-1222.1

14. Multi-Resolution Land Characteristics (MRLC) Consortium | Multi-Resolution Land Characteristics (MRLC) Consortium. [cited 20 Jul 2019]. Available: https://www.mrlc.gov/

15. EarthExplorer - Home. [cited 20 Jul 2019]. Available: https://earthexplorer.usgs.gov/

16. Gartner MH, Veblen TT, Sherriff RL, Schoennagel TL. Proximity to grasslands influences fire frequency and sensitivity to climate variability in ponderosa pine forests of the Colorado Front Range. Int J Wildland Fire. 2012;21: 562–571. doi:10.1071/WF10103

17. Dwire KA, Kauffman JB. Fire and riparian ecosystems in landscapes of the western USA. Forest Ecology and Management. 2003;178: 61–74. doi:10.1016/S0378-1127(03)00053-7

18. Pettit NE, Naiman RJ. Fire in the Riparian Zone: Characteristics and Ecological Consequences. Ecosystems. 2007;10: 673–687. doi:10.1007/s10021-007-9048-5

19. Wilkin KM, Ackerly DD, Stephens SL. Climate Change Refugia, Fire Ecology and Management. Forests. 2016;7: 77. doi:10.3390/f7040077

20. Whitman E, Parisien M-A, Thompson DK, Hall RJ, Skakun RS, Flannigan MD. Variability and drivers of burn severity in the northwestern Canadian boreal forest. Ecosphere. 2018;9: e02128. doi:10.1002/ecs2.2128

21. NHD Plus - NHDPlus Version 2. [cited 20 Jul 2019]. Available: http://www.horizon-systems.com/nhdplus/NHDPlusV2_home.php

22. Donchyts G, Winsemius H, Schellekens J, Erickson T, Gao H, Savenije H, et al. Global 30m Height Above the Nearest Drainage. 2016. doi:10.13140/RG.2.1.3956.8880

23. LANDFIRE. [cited 1 May 2015]. Available: http://www.landfire.gov

24. Bigio ER, Swetnam TW, Baisan CH. Local-scale and regional climate controls on historical fire regimes in the San Juan Mountains, Colorado. Forest Ecology and Management. 2016;360: 311–322. doi:10.1016/j.foreco.2015.10.041

25. Dillon Gregory K., Holden Zachary A., Morgan Penelope, Crimmins Michael A., Heyerdahl Emily K., Luce Charles H. Both topography and climate affected forest and woodland burn severity in two regions of the western US, 1984 to 2006. Ecosphere. 2011;2: art130. doi:10.1890/ES11-00271.1

26. Camp A, Oliver C, Hessburg P, Everett R. Predicting late-successional fire refugia pre-dating European settlement in the Wenatchee Mountains. Forest Ecology and Management. 1997;95: 63–77. doi:10.1016/S0378-1127(97)00006-6

27. Theobald DM, Harrison-Atlas D, Monahan WB, Albano CM. Ecologically-Relevant Maps of Landforms and Physiographic Diversity for Climate Adaptation Planning. PLOS ONE. 2015;10: e0143619. doi:10.1371/journal.pone.0143619

28. Abella SR, Denton CW. Spatial variation in reference conditions: historical tree density and pattern on a Pinus ponderosa landscape. Can J For Res. 2009;39: 2391–2403. doi:10.1139/X09-146

29. Keith RP, Veblen TT, Schoennagel TL, Sherriff RL. Understory vegetation indicates historic fire regimes in ponderosa pine-dominated ecosystems in the Colorado Front Range. Journal of Vegetation Science. 2010;21: 488–499. doi:10.1111/j.1654-1103.2009.01156.x

30. Chaney NW, Wood EF, McBratney AB, Hempel JW, Nauman TW, Brungard CW, et al. POLARIS: A 30-meter probabilistic soil series map of the contiguous United States. Geoderma. 2016;274: 54–67. doi:10.1016/j.geoderma.2016.03.025

31. Meddens AJH, Kolden CA, Lutz JA, Smith AMS, Cansler CA, Abatzoglou JT, et al. Fire Refugia: What Are They, and Why Do They Matter for Global Change? BioScience. 2018 [cited 9 Oct 2018]. doi:10.1093/biosci/biy103

32. Cansler CA, McKenzie D. Climate, fire size, and biophysical setting control fire severity and spatial pattern in the northern Cascade Range, USA. Ecological Applications. 2014;24: 1037–1056. doi:10.1890/13-1077.1
