# Supplementary material for: Still standing: Recent patterns of post-fire conifer refugia in ponderosa pine-dominated forests of the Colorado Front Range
Source: PLoS One. 2020 Jan 15;15(1):e0226926. doi: 10.1371/journal.pone.0226926 (PMC6961861; doi:10.1371/journal.pone.0226926)
Supplement: S4 Table — Maximum, mean, and standard deviation of distance to potential pre- and post-fire seed source for the 23 fires that burned ponderosa pine-dominated forests along Colorado’s Front Range 1996–2013. (DOCX) [file pone.0226926.s004.docx]

|  | Pre-fire Distance to Seed Source | | | Post-fire Distance to Seed Source | | |
| --- | --- | --- | --- | --- | --- | --- |
| Fire (Year Fire Name) | Maximum | Mean | STD | Maximum | Mean | STD |
| 1996 Buffalo Creek | 60 | 0.1 | 1.6 | 517 | 84.1 | 92.6 |
| 2000 Bobcat | 67 | 0.4 | 3.7 | 692 | 60.5 | 97.1 |
| 2000 Eldorado/Walker Ranch | 67 | 0.7 | 4.9 | 182 | 17.7 | 29.1 |
| 2000 High Meadow | 85 | 0.3 | 3.1 | 591 | 45.9 | 74.3 |
| 2002 Big Elk | 42 | 0.5 | 4.0 | 268 | 24.0 | 40.5 |
| 2002 Hayman | 108 | 0.4 | 3.6 | 1291 | 111.0 | 167.0 |
| 2002 Schoonover | 60 | 0.4 | 3.3 | 416 | 71.6 | 82.2 |
| 2002 Spring | 150 | 0.8 | 5.4 | 842 | 73.2 | 125.6 |
| 2003 Overland | 60 | 0.6 | 4.2 | 593 | 71.6 | 100.9 |
| 2004 Picnic Rock | 306 | 20.7 | 37.9 | 323 | 42.5 | 50.0 |
| 2005 Mason | 124 | 0.6 | 4.8 | 1140 | 160.1 | 197.0 |
| 2006 Mato Vega | 180 | 4.1 | 13.1 | 474 | 55.1 | 74.2 |
| 2006 Mauricio Canyon | 134 | 7.6 | 16.3 | 510 | 89.1 | 103.7 |
| 2010 Four Mile Canyon | 90 | 2.4 | 8.7 | 436 | 38.9 | 67.3 |
| 2011 Crystal | 67 | 2.9 | 9.6 | 335 | 53.0 | 67.3 |
| 2011 Indian Gulch | 95 | 8.9 | 15.3 | 134 | 22.5 | 26.1 |
| 2012 Hewlett | 212 | 9.6 | 24.4 | 713 | 58.5 | 100.1 |
| 2012 High Park | 218 | 2.2 | 9.5 | 1176 | 91.4 | 139.5 |
| 2012 Lower North Fork | 134 | 2.7 | 10.8 | 391 | 54.3 | 73.9 |
| 2012 Springer | 42 | 1.0 | 5.4 | 301 | 21.0 | 48.5 |
| 2012 Waldo Canyon | 228 | 3.5 | 13.1 | 706 | 66.8 | 105.4 |
| 2012 Wetmore | 95 | 5.2 | 12.6 | 313 | 46.3 | 60.0 |
| 2013 East Peak | 90 | 2.9 | 9.7 | 361 | 31.1 | 49.2 |
