# Supplementary figures and images for: Still standing: Recent patterns of post-fire conifer refugia in ponderosa pine-dominated forests of the Colorado Front Range
Source: PLoS One. 2020 Jan 15;15(1):e0226926. doi: 10.1371/journal.pone.0226926 (PMC6961861; doi:10.1371/journal.pone.0226926)

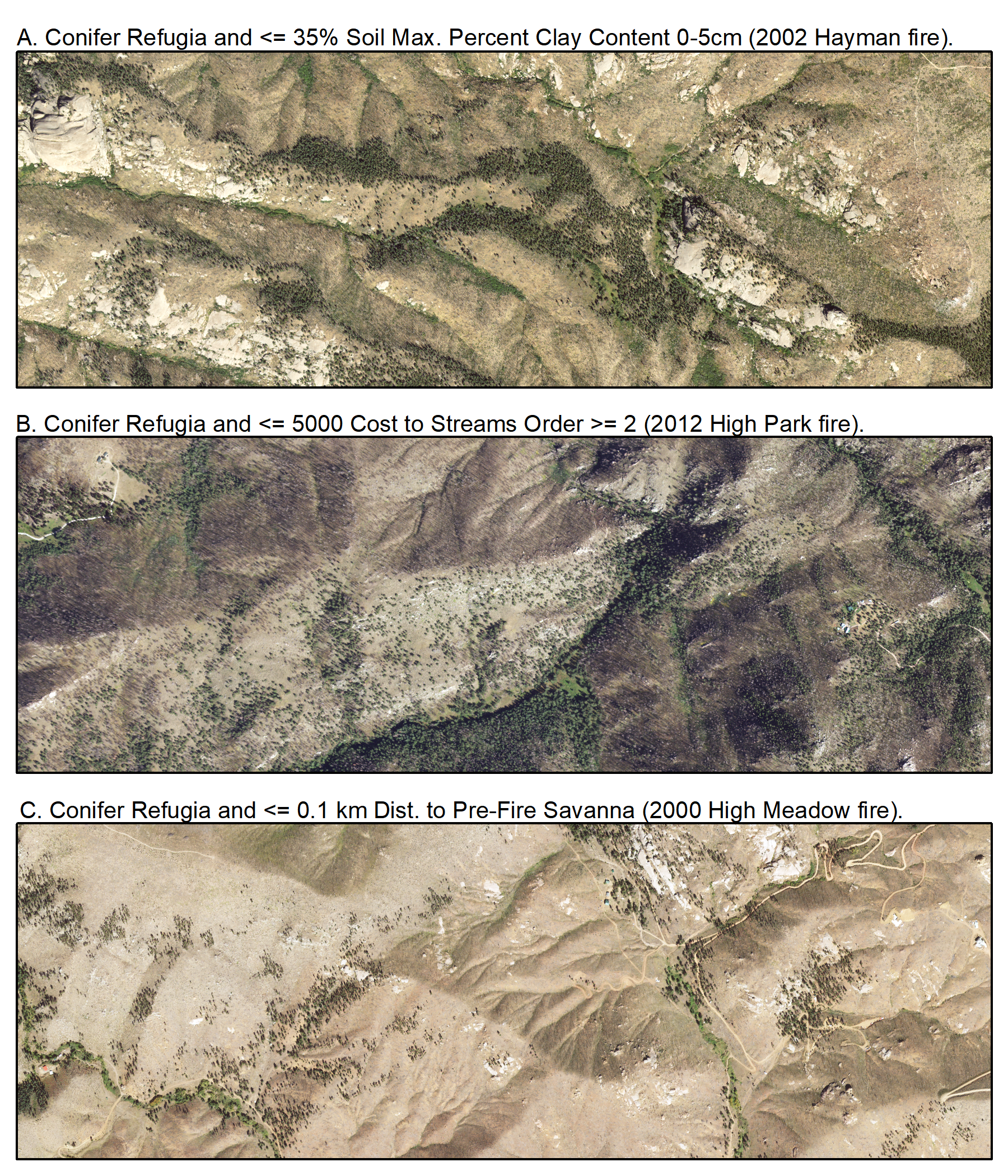

Supplement: S1 Fig — Three examples of Conifer Refugia within 23 fires that burned ponderosa pine-dominated forests along Colorado’s Front Range 1996–2013 as shown by 2015 NAIP aerial imagery. (TIF) [file pone.0226926.s005.tif]
