# Supplementary material for: Still standing: Recent patterns of post-fire conifer refugia in ponderosa pine-dominated forests of the Colorado Front Range
Source: PLoS One. 2020 Jan 15;15(1):e0226926. doi: 10.1371/journal.pone.0226926 (PMC6961861; doi:10.1371/journal.pone.0226926)
Supplement: S1 Text — (DOCX) [file pone.0226926.s006.docx]

To generate daily fire weather variables, we determined burn dates for each pixel using three sources: 1) GeoMAC Fire perimeters, 2) MODIS Burned Area Products, and 3) FEMA incident reports. GeoMAC fire perimeters are created by a USGS fire support program that collects near-daily information on fire growth from incident intelligence sources, GPS data, as well as infrared (IR) imagery from fixed wing and satellite platforms [1]. The MODIS Burned Area Products use 500 m MODIS satellite imagery to detect changes in reflectance based on loss of vegetation and occurrence of ash [2]. For every fire, we gathered beginning and ending burn dates from FEMA’s fire incident reports, which document the incident dates for disasters and the Fire Management Assistance Declaration for fires [3]. All burn dates were converted to Julian calendar dates and pixels were assigned to the first recorded date in either GeoMAC or MODIS Burned Area Products. Burn dates that were either before or after the FEMA dates were reassigned to either the first or last date of the fire respectively. For fires not recorded by GeoMAC or MODIS Burned Area Products, we assigned the entire fire to the date FEMA declared Fire Management Assistance Declaration as this is assumed to be the date the fire experienced initial spread. For each burn date, we downloaded daily fire weather (maximum wind speed, maximum temperature, and minimum relative humidity) from the closest USFS Remote Automated Weather Station (RAWS) [4]. We used the archived USFS Wildland Fire Assessment System’s Fire Danger Rating to reflect daily burning conditions [5]. This standardized ranking (1-5: low, moderate, high, very high, extreme) utilizes local current and antecedent weather and fuels (fuel type, and live and dead fuel moisture) information formulated into a Burning Index and Energy Release Component interpolated at a 10-km resolution between weather stations. Seven fire days did not have Fire Danger Rating data and were assigned the same rating as either the previous or following day based on similarity of RAWS data for those days.

We used the 2001 National Land Cover Database (NLCD) percent tree canopy as the foundation for the Pre-fire Forest Cover layer. This continuous layer (0-100%) is created from Landsat Thematic Mapper images in 2000 at a 30 m resolution and covered all fires except the 1996 Buffalo Creek fire. The 2001 NLCD tree cover estimates generally underestimates tree cover on average 9.7% across the US [6]. Ten percent of the surviving trees (Conifer Refugia) occurred in areas zero percent tree canopy cover pre-fire, as classified by the 2001 NLCD percent tree canopy. Therefore, we improved the zero percent tree canopy class by using 1999 1m Digital Orthophoto Quadrangles (DOQs) as additional tree cover information. We created a binary threshold classification of shadow (reflectance < 125) to identify trees in grid cells of zero canopy cover in the 2001 NLCD percent tree canopy. We summed the number of 1-m pixels classified as dark in a 30-m grid cell and reclassified all pixels with between one and 30% dark as <10% tree cover, assuming large areas of dark reflectance were cliffs or wet meadows. For the Buffalo Creek fire, we obtained three panchromatic 1994 National Aerial Photography Program (NAPP) images and georeferenced them at a 1:52000 scale using the center point of the image and reference points from the 1999 DOQs. Following georeferencing, these images were resampled to 1m resolution to align with NAIP imagery. We used two steps to classify forests in NAPP aerial imagery: 1) a binary threshold of shadow/tree and non-forest to classify Pre-fire Forest Cover (reflectance < 95) and, 2) a texture measure using a standard deviation of reflectance of less than 15 and a reflectance of less than 120 in an 11x11 window. The second step captures very dense stands that do not create dark shadows captured in the first step. The final Pre-fire Forest Cover variable included the 2001 NLCD percent tree canopy, the modified 1-10% tree canopy cover from the 1999 classified DOQs, and the 1994 Buffalo Creek Pre-fire Forest Cover. Pre-fire Forest Cover was binned to 10% increments for analysis.

**References**

1. GeoMAC Wildfire Application [Internet]. [cited 19 Jul 2019]. Available: https://www.geomac.gov/

2. Roy DP, Boschetti L, Justice CO, Ju J. The collection 5 MODIS burned area product — Global evaluation by comparison with the MODIS active fire product. Remote Sensing of Environment. 2008;112: 3690–3707. doi:10.1016/j.rse.2008.05.013

3. Disasters | FEMA.gov [Internet]. [cited 19 Jul 2019]. Available: https://www.fema.gov/disasters

4. RAWS USA Climate Archive State Selection Map [Internet]. [cited 19 Jul 2019]. Available: https://raws.dri.edu/

5. Fire Danger Rating [Internet]. [cited 19 Jul 2019]. Available: https://www.wfas.net/index.php/fire-danger-rating-fire-potential--danger-32

6. Greenfield EJ, Nowak DJ, Walton JT. Assessment of 2001 NLCD Percent Tree and Impervious Cover Estimates. Photogrammetric Engineering & Remote Sensing. 2009;75: 1279–1286. doi:10.14358/PERS.75.11.1279
